# Association of maternal gut microbiota and plasma metabolism with congenital heart disease in offspring: a multi-omic analysis

**DOI:** 10.1038/s41598-021-84901-7

**Published:** 2021-03-05

**Authors:** Tingting Wang, Lizhang Chen, Peng Huang, Tubao Yang, Senmao Zhang, Lijuan Zhao, Letao Chen, Ziwei Ye, Liu Luo, Jiabi Qin

**Affiliations:** 1 NHC Key Laboratory of Birth Defect for Research and Prevention, Hunan Provincial Maternal and Child Health Care Hospital, Changsha, Hunan China; 2 Department of Epidemiology and Health Statistics, Xiangya School of Public Health, Central South University, Changsha, Hunan China; 3 Hunan Provincial Key Laboratory of Clinical Epidemiology, Changsha, Hunan China; 4 Department of Thoracic Cardiac Surgery, Hunan Children’s Hospital, Changsha, Hunan China; 5 Guangdong Cardiovascular Institute, Guangdong Provincial People’s Hospital, Guangdong Academy of Medical Sciences, Guangzhou, Guangdong China

**Keywords:** Heart development, Microbial genetics, Diseases

## Abstract

Congenital heart disease (CHD) is the most common congenital disorder diagnosed in newborns. Although lots of related studies have been published, yet the pathogenesis has not been fully elucidated. A growing body of evidence indicates perturbations of the gut microbiota may contribute in a significant way to the development of obesity and diabetes. Given that maternal obesity and diabetes are well-known risk factors for CHD, maternal gut microbiota may be considered as one of the environmental factors involved in the pathogenesis of CHD. The object of this study is to explore the association between maternal gut microbiota and risk of congenital heart disease (CHD) in offspring, as well as the possible mechanisms linking gut microbiota and disease risk. A case–control study was conducted in mothers of infants with CHD (n = 101) and mothers of infants without CHD (n = 95). By applying 16S rRNA gene sequencing and metabolic approaches to 196 stool and plasma samples, we determined microbiome and metabolome profiles in mothers of infants with CHD and controls, and their association with risk of CHD in offspring. The gut microbiome of mothers of infants with CHD was characterized with lower alpha-diversity and distinct overall microbial composition compared with mothers of infants without CHD. A distinct different metabolic profile was found between mothers of infants with CHD and controls. After controlling for the possible confounders, thirty-four bacterial genera and fifty-three plasma metabolites showed distinct abundances between the two groups. The results of the Spearman correlation analyses revealed a great number of significant correlations between the abundant bacterial genera and differentially expressed metabolites. In particular, the genus *Bifidobacterium* and *Streptococcus* showed comparable moderate positive correlations with a range of metabolites that involved in lipid metabolism pathway. Our findings suggest that perturbations of maternal gut microbiota and plasma metabolites may be associated with risk of CHD in offspring, and co-variation between microbiota and metabolites may play a part in the linkage between gut microbiota and risk of CHD in offspring.

## Introduction

Nowadays, congenital heart disease (CHD) has emerged as the single largest cause of infant morbidity and mortality worldwide^1^. Furthermore, CHD have a significant impact on child and adult morbidity^2–4^. Over the last two decades, major breakthroughs have been made in the understanding of risk factors for CHD, especially for the identification of specific genetic abnormalities in certain CHD phenotypes. However, little information is available about the modifiable environmental factors which may have adverse effects on fetal cardiac development. To date, only a few known risk factors, such as maternal pregestational obesity and diabetes, have been identified. The limited understanding of modifiable risk factors of CHD has hampered its prevention.

With the rapid development of the next generation sequencing (NGS), the gut microbiota as an environmental factor has attracted considerable attention from scientists and clinicians. It has been well confirmed that the gut microbiota can produce a variety of compounds that regulate the activity of the distal organs and play an important role in the host metabolism, nutrient absorption, and immunity. The composition of the gut microbiome can be readily affected by extrinsic factors such as antibiotics, diet, lifestyle, and body mass index^5,6^, and 57% of the structural variation in gut microbiota can be attribute to diet changes^7^. A growing body of evidence indicates that perturbations of the gut microbiota and its influence on metabolic and physiological functions may contribute in a significant way to the development of human diseases such as obesity and diabetes^8,9^. Given that maternal obesity and diabetes prior to pregnancy are well-known risk factors for CHD^10,11^, preconception maternal gut microbiota may be considered as one of the environmental factors involved in the pathogenesis of CHD.

Evidences suggest that the host metabolism is regulated by its own genome and the commensal gut microbial genome, and the host’s metabolic phenotype represents an amalgamation of human and microbial attributes^12,13^. The gut microbiota interacts extensively with the host through substrate co-metabolism and metabolic exchange, and has a co-metabolism interaction with the host. The application of the NGS technology has deepened our knowledge about the taxonomic profile of the microbial community and its correlation with human health; however, the identification of gut microbes which play a critical role in the modulation of the host’s metabolic profiles, as well as their mode of action, cannot be achieved by NGS alone. An integrated approach combining NGS and metabolomics can overcome the limitations in researches based only on genomics. System biology approaches that combine metabolomics with other “omics” researches have the potential to provide a comprehensive picture of complex diseases.

As mentioned earlier, accumulating evidences demonstrate that gut microbiota has contributed to the developmental of obesity and diabetes. Given that obesity and diabetes occurred before pregnancy in women have been confirmed as important risk factors for CHD, it is suggested that alterations in maternal gut microbiota may be associated with the developmental of CHD in offspring. Considering that the key development phase of heart is the third to eighth gestational weeks, a prospective preconception cohort study is the most effective way to investigate the association between maternal gut microbiota and risk of CHD. However, it is not practicable to conduct a preconception cohort of CHD considering its relatively low birth prevalence rate as well as the high financial costs. Pieces of evidences suggest that at 1-year postpartum, dietary patterns and lifestyle of the parous women, which are important external impact factors of gut microbiome, has gotten back to the status prior to pregnancy^14–16^. Therefore, as an alternative, a case–control study which regards 1 year after delivery of parous women as a starting point is valuable to a certain degree for the preliminary exploration of the association of maternal gut microbiota with CHD.

From this background, a case–control study was conducted in mothers of infants with CHD and their controls. An integrated approach combining 16S rRNA gene sequencing and plasma metabolomics was applied to analyze both the maternal gut microbiome and plasma metabolic profiles, and to explore their association with risk of CHD in offspring.

## Results

### Characteristics of the study population

A total of 196 participants were enrolled at Hunan Children’s Hospital, including 101 mothers of infants with CHD (MCHD) and 95 mothers of infants without CHD (MCG). The main characteristics of the 196 subjects as well as the extrinsic host factor profiles including lifestyle and diet were summarized in Supplementary Table 1. The distribution of ethnicity was significantly different between mothers of infants with CHD and mothers of infants without CHD, while that of characteristics including age, BMI, residence was comparable between the two groups (all p > 0.05). While one mother of infant with CHD reported a history of type II diabetes, none of the others reported a history of hyperglycemia, hypertension, hyperlipoidemia, hyperuricacidemia, phenylketonuria, or hyperhomocysteinemia. Only a small percentage of participants reported smoking or drinking behaviors, and this percentage did not differ between mothers of infants with CHD and the control group (all p > 0.05). The proportions of negative life events in both groups were also consistent. With regard to diet, the intake frequencies of water products, eggs, and beans/bean products intake were significantly different between the two groups (all p > 0.05). In addition, the main characteristics of infants of the participants were also summarized in Supplementary Table 1. Specifically, the distribution of gender of infants was significantly different between mothers of infants with CHD and the control group with a higher proportion of male infants in the control group (p = 0.026).

### Decreased alpha-diversity and altered overall microbial composition in mothers of infants with CHD

**Figure 1:**
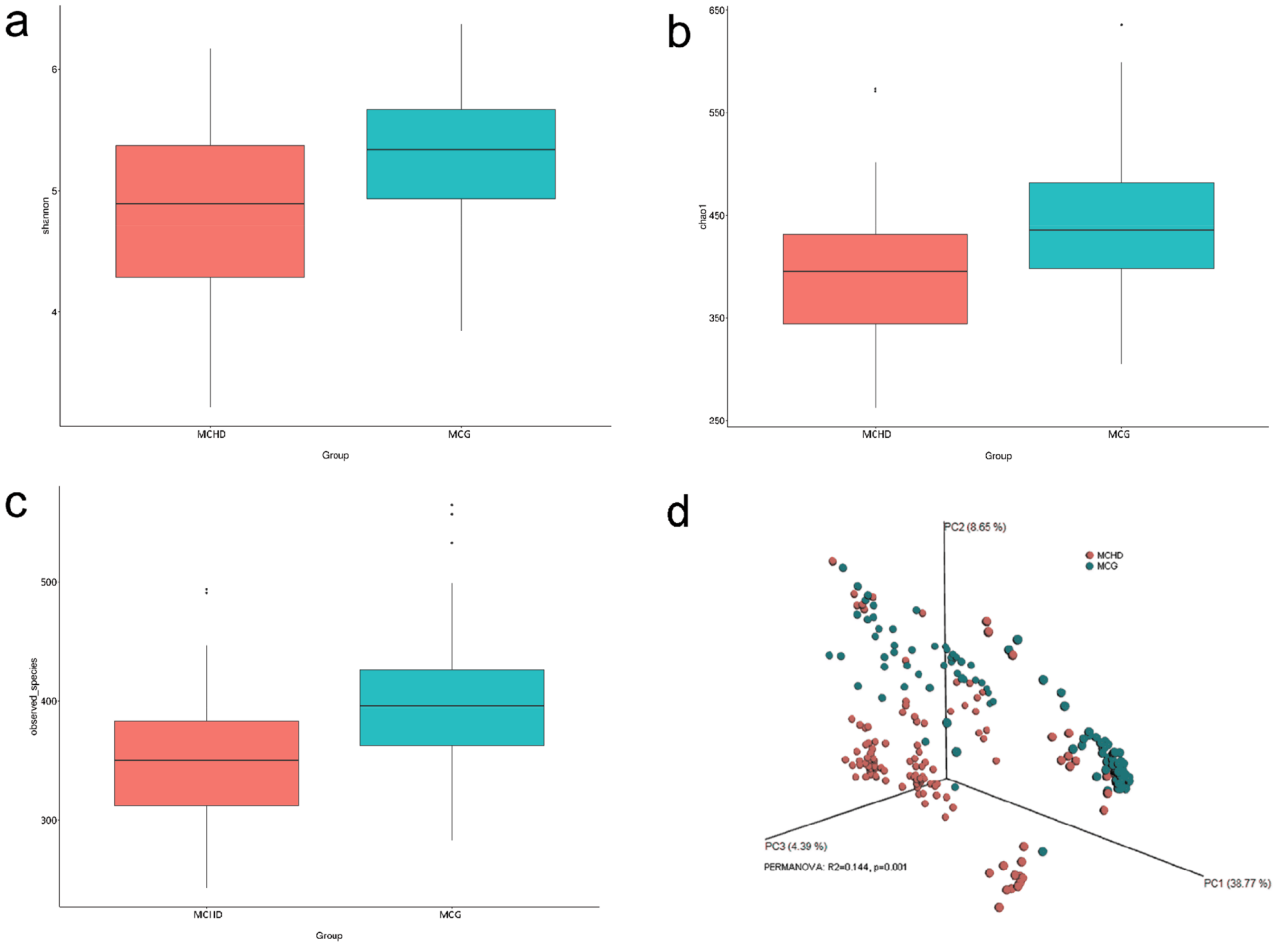
Comparisons of gut microbial alpha-diversity and beta-diversity between mothers of infants with CHD and mothers of infants without CHD. Compared with the controls, gut microbial diversity, as estimated by the Shannon index (**a**), Chao1 index (**b**), and the observed species (**c**), was significantly decreased in mothers of infants with CHD (all p < 0.05). (**d**) PCoA based on unweighted UniFrac distance showed that the overall gut microbiota composition was different between mothers of infants with CHD and controls (R^2^ = 0.144, p = 0.001). *CHD* congenital heart disease, *PCoA* principal coordinates analysis.

In the present study, we compared the gut microbiota of mothers of infants with CHD with that of mothers of infants without CHD by next-generation sequencing of the 16S rRNA gene. After sequencing and quality filtering, a total number of 14,616,519 reads with an average length of 256.34 base pairs were obtained corresponding to a mean of 74,574 reads per sample. A total of 2858 OTUs were clustered by reads at 97% identity. By measuring alpha diversity using the Shannon index, Chao1 index, and the observed_species, we found that mothers of infants with CHD had significantly decreased microbial diversity and evenness in comparison with their controls (Fig. 1a–c, all p < 0.05; Supplementary Fig. 1). All findings were still significant after adjusting for covariates including age, BMI, ethnicity, residence, cigarette smoking, alcohol consumption, negative life events, and dietary intake (Supplementary Table 2).

Beta diversity was calculated using both unweighted and weighted UniFrac distance matrices. The top three components of PCoA based on unweighted UniFrac distances were plotted (Fig. 1d). PERMANOVA analysis showed a significant difference, with respect to overall phylogenetic distance of gut microbiome composition, between mothers of infants with CHD and the control group (p = 0.001, Fig. 1d); the difference was still statistically significant after adjusting for those covariates mentioned above (p = 0.001, Supplementary Table 3). The microbial community between the two groups was also significantly different basing on weighted UniFrac distance (p = 0.001, Supplementary Fig. 2).

### Microbiota differentially abundant in mothers of infants with CHD versus controls

**Figure 2:**
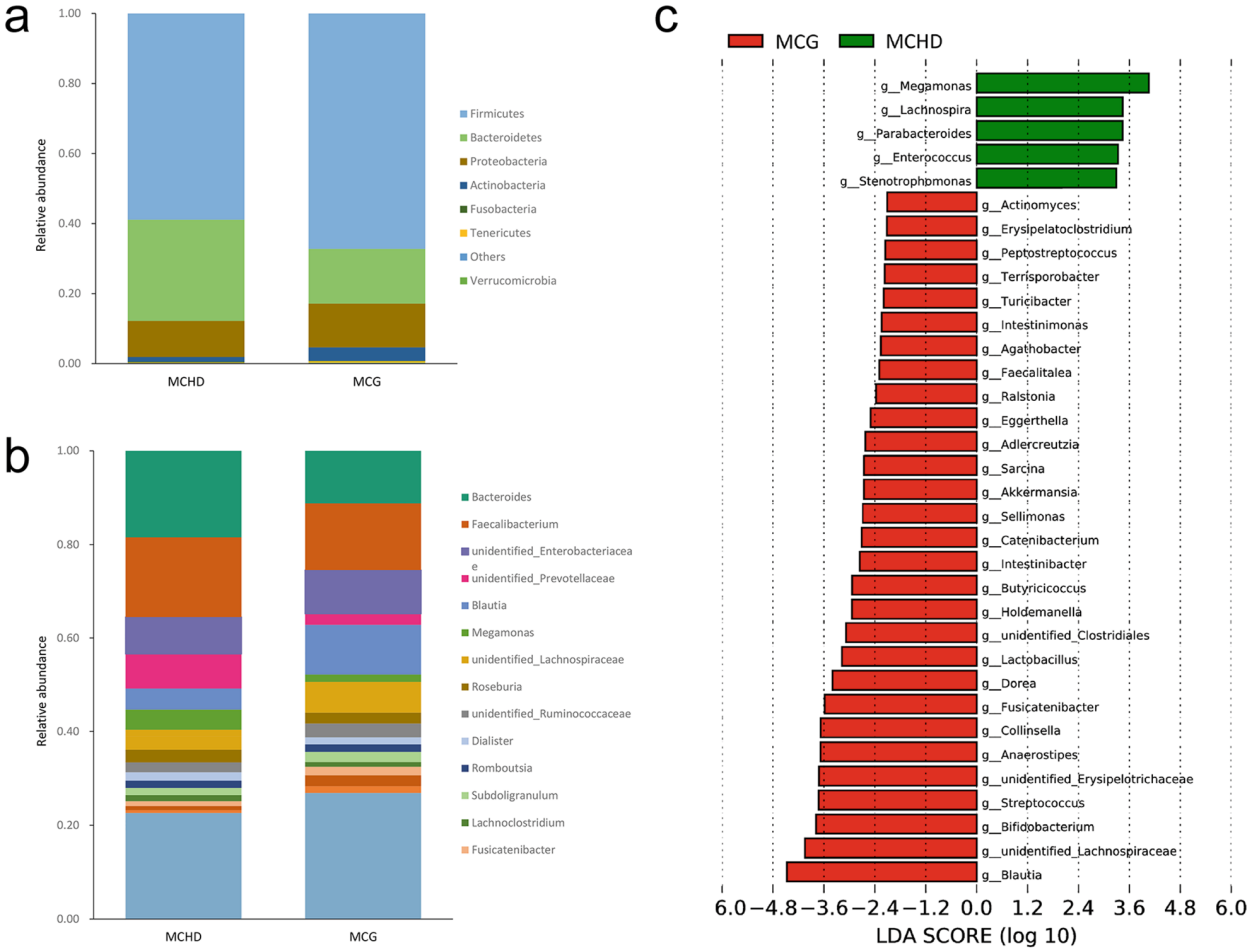
Variations of gut microbiota composition in mothers of infants with CHD. Relative proportions of bacterial taxa at the phylum level (**a**) and genus level (**b**) were different between mothers of infants with CHD and controls. Only those bacterial phyla and genera with a relative abundance > 1% were presented independently, the others were merged and presented in “others”. (**c**) LDA score of thirty-four differentially abundant genera which were identified by LEfSe analysis and MaAsLin. *CHD* congenital heart disease, *LDA* line discriminant analysis, *MaAsLin* Multivariate Association with Linear Models algorithm.

To identify differentially abundant taxa, LEfSe analysis on the gut microbiota composition was performed. *Firmicutes* and *Bacteroidetes* were the most dominant phylum in both mothers of infants with CHD and the control group. Significant changes were observed at the phylum level between the two groups (Fig. 2a). At the genus level, significant differences were observed between mothers of infants with CHD and controls (Fig. 2b). A total of 219 bacterial taxa showed distinct relative abundances between the two groups, out of which 86 taxa at the genus level were significantly enrich in mothers of infants with CHD or the control group (LDA score > 2, Q_FDR_ < 0.05; Supplementary Table 4). Thirty-four of these genera remained significantly different after a multivariate analysis (MaAsLin) was performed to control the possible influence of age, BMI, ethnicity, residence, cigarette smoking, alcohol consumption, negative life events, and dietary intake (Fig. 2c). Decreased abundance in 29 genera and increased abundance in 5 genera were observed in mothers of infants with CHD.

### Changes in the plasma metabolomic features between mothers of infants with CHD and controls

**Figure 3:**
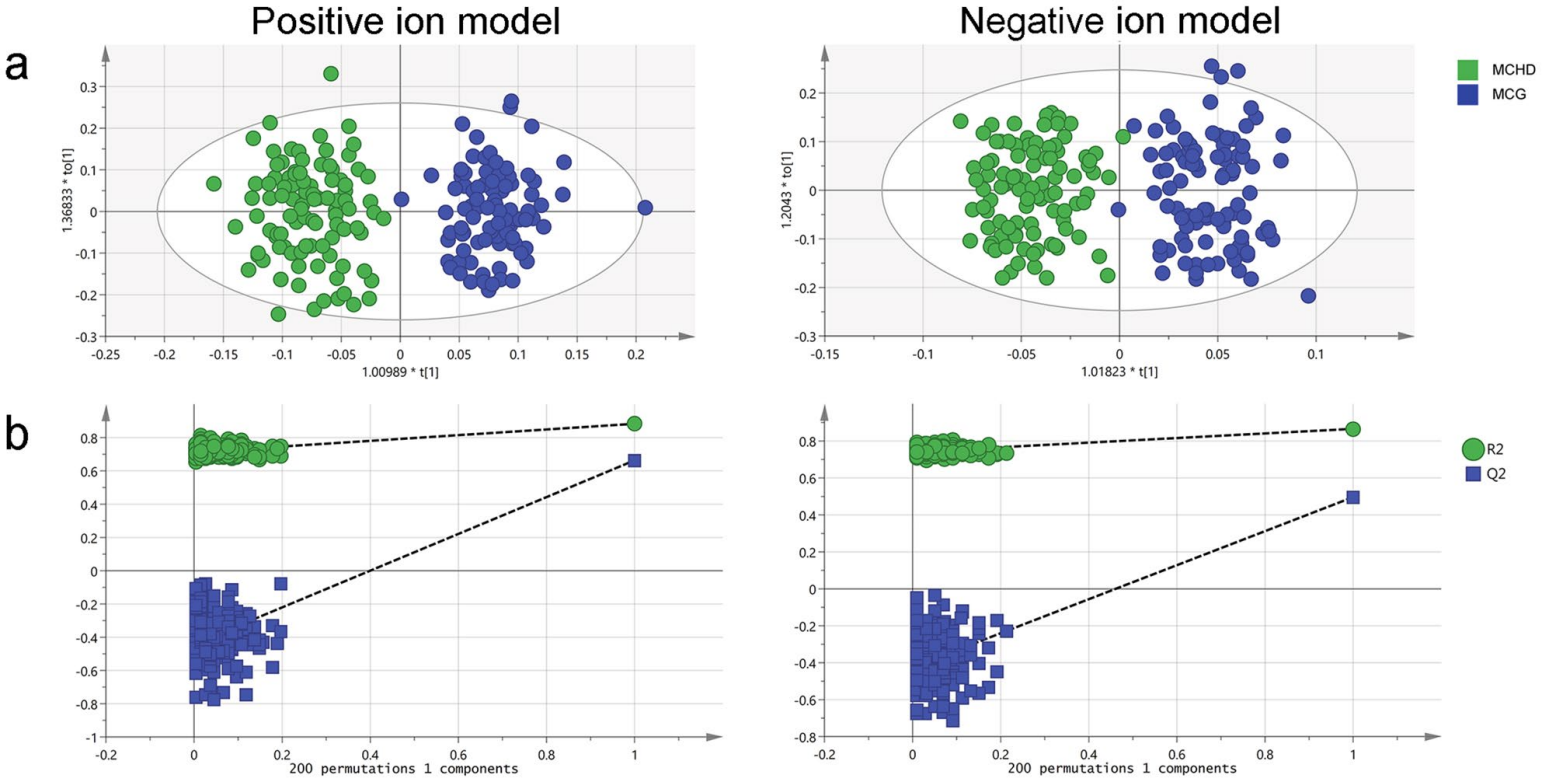
Alteration of metabolites between mothers of infants with CHD and controls. (**a**) OPLS-DA of plasma samples from mothers of infants with CHD and controls under positive and negative ion mode (positive ion model: R2X(cum) = 0.248, R2Y(cum) = 0.977, Q2(cum) = 0.752; negative ion model: R2X(cum) = 0.234, R2Y(cum) = 0.949, Q2(cum) = 0.683). (**b**) Validation of OPLS-DA model under positive and negative ion mode (using 200 random permutations). *CHD* congenital heart disease, *OPLS-DA* orthogonal partial least squares-discrimination analysis.

To identify the plasma metabolomic features of mothers of infants with CHD, untargeted metabolome profiles were generated on samples from mothers of infants with CHD and their controls by UPLC-MS. Metabolomic profiling yielded 7260 features. In the OPLS-DA model of metabolite profiling data (Fig. 3 and Supplementary Fig. 3), there was a significant difference in the metabolic phenotype between mothers of infants with CHD and the control group, suggesting that a distinct metabolic profile might exist in mothers of infants with CHD (Fig. 3a). The results of the permutation test with 200 iterations indicated the reliability of the model (Fig. 3b). With a significance level of Q_FDR_ < 0.05 for the primary composition between mothers of infants with CHD and their controls, 58 metabolites were found to be significantly different (Supplementary Table 5 and Supplementary Fig. 4). After controlling for covariates including age, BMI, ethnicity, residence, cigarette smoking, alcohol consumption, negative life events, and dietary intake, 53 metabolites were still significantly different between the two groups and were regarded as differentially expressed metabolites (p < 0.05; Supplementary Table 5). Among those differentially expressed metabolites, 28 metabolites were increased in mothers of infants with CHD.

Moving beyond the analysis of individuals metabolites, KEGG analysis was performed to investigate the metabolic pathways significantly associated with the disease status of the offspring according to the differentially expressed metabolites. Results showed that these differential metabolites were enriched 19 metabolic pathways (Supplementary Fig. 5), out of which choline metabolism in cancer pathway was the most significantly associated with the disease status of the offspring.

### Distinct correlations between abundant bacterial genera and significant metabolites

**Figure 4:**
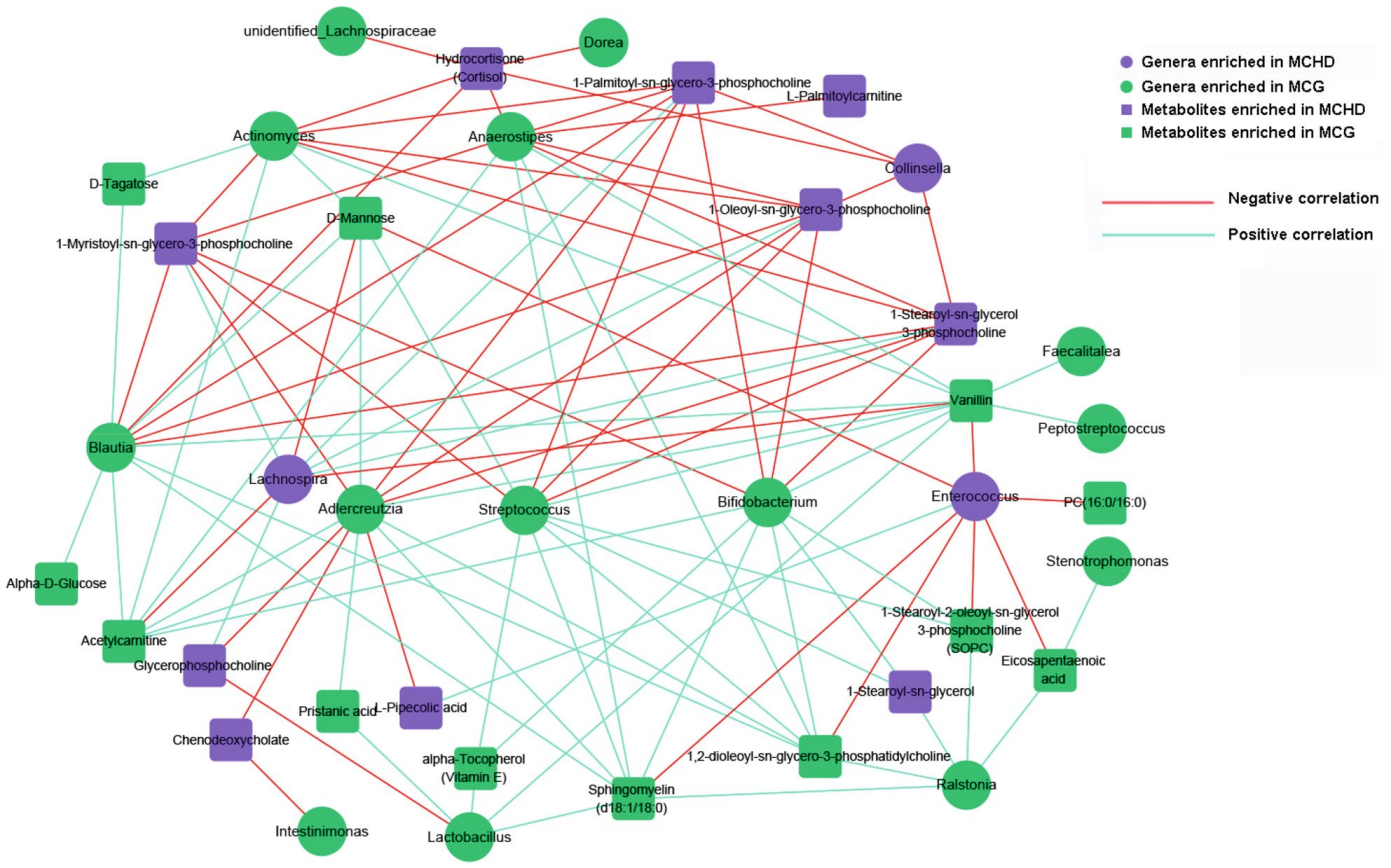
Microbiota-metabolites correlation network based on Spearman’s correlation coefficients. Each node represents one species (circle) or metabolite (square), and two nodes are linked if the correlation was significant (two-sided pseudo p < 0.05) and more than 0.3. Lines between nodes show positive correlations (blue lines) or negative correlations (red lines). The node size is proportional to the number of correlated metabolites.

Multi-omic analysis was performed using Spearman correlation coefficients to determine the correlations between the abundant bacterial genera and differentially expressed metabolites. A total of 1,802 correlation coefficient of microbiota-metabolites pairs were computed, out of which 747 were statistically significant (Q_FDR_ < 0.05; Supplementary Fig. 6). Under the correlation cut-off with 0.3, 42 nodes (19 genera and 23 metabolites) and 129 edges (connections) were retained in the correlation network plot (Fig. 4). *Bifidobacterium* and *Streptococcus* showed comparable moderate positive correlations with a range of metabolites that were significantly increased in controls, including Sphingomyelin (d18:1/18:0), 1,2-dioleoyl-sn-glycero-3-phosphatidylcholine, 1-Stearoyl-2-oleoyl-sn-glycerol 3-phosphocholine (SOPC), 1-Stearoyl-sn-glycerol, Acetylcarnitine, alpha-Tocopherol (Vitamin E), and Vanillin; except for Vanillin mapped to phenylalanine metabolism pathway, the remaining six metabolites were involved in lipid metabolism pathway. In addition, *Lactobacillus*, *Anaerostipes*, and *Adlercreutzia* also showed moderate correlations with part of those metabolites.

## Discussion

In the current study, an integrated 16S rRNA sequencing and UPLC-MS based metabolomics approach was performed to explore the possible associations between maternal gut microbiome and plasma metabolites and risk of CHD in offspring. The results showed that mothers of infants with CHD had significantly different gut microbiota and plasma metabolite profiles compared with their controls. Through multi-omics analyses, our study found a number of associations between the altered gut microbiota and plasma levels of metabolites, suggesting that maternal metabolic phenotype correlates with gut microbiota composition and that this might be associated with the risk of CHD in offspring. To our knowledge, this is the first report concerning the gut microbiota composition and plasma metabolite profile in mothers of infants with CHD, although we are aware of the fact that owing to the various limitations in this study, one should regard this report as a preliminary observation in an important filed. The results of this study can provide novel clues and directions, from the perspective of maternal gut microbiota, for further researches concerning the potential etiology and preventive target for CHD.

The gut microbiota living in the digestive tract is composed of a diverse and complex community of microorganisms, which have established a symbiotic relationship with humans in the course of evolution. In general, the human gut microbiota exists as an ecosystem in which individuals interact and balance with each other to form a stable system, and plays an important role in immunity, metabolism, and nutrition absorption. Probiotics, such as *Bifidobacterium* and *Lactobacillus*, are involved in molecular crosstalk with intestinal epithelium and affect intestinal barrier function. In this study, using 16S rRNA sequencing, the abundance of *Bifidobacterium* and *Lactobacillus* were found to be markedly decreased in mothers of infants with CHD after controlling for covariates; this may weaken the physiological barrier function of the gut mucosa by increasing its permeability, and promote bacterial translocation^17^. The lipopolysaccharide (LPS) released by bacterial lysis enters the blood through the intestinal epithelium with increased permeability, and then, binds to the LPS-binding protein to cause the inflammatory cascade reaction in the host^18^. One the one hand, this chronic inflammation may cause vascular endothelial dysfunction, increase systemic vascular resistance, and then elevate blood pressure^19,20^. Elevated blood pressure in the mother can lead to uteroplacental insufficiency that may result in compromised blood flow to the developing fetus, which, if present in the third to eighth gestational age, may increase the risk of cardiovascular malformations^21,22^. On the other hand, the inflammatory reaction caused by gut microbiota perturbations can stimulate the production of inflammatory factors which may induce insulin resistance by affecting signal transduction pathway of insulin, such as insulin receptor substrate phosphorylation^23^. Given that insulin and related signaling pathways have been proven to be key mediators of embryogenesis and early development, maternal gut microbiota perturbations may exert a teratogenic effect through a signaling pathway regulating insulin sensitivity^24,25^. Furthermore, *Bifidobacterium* and *Lactobacillus* have been proposed as possible folate producers^26,27^. Evidences suggest that folate may play a role in the migration of cardiac nerve cells, which contributes to the development of the embryonic heart^28^. When maternal folate supplementation and dietary folate intake is insufficient, a decrease in the abundance of *Bifidobacterium* and *Lactobacillus* may result in a decrease in maternal circulating levels of folate which may eventually affect the development of the embryonic heart.

In the present study, metabolic phenotypes revealed significant differences between mothers of infants with CHD and the control group, suggesting that maternal metabolic changes may be associated with risk of CHD in offspring. Especially, the plasma level of sphingomyelin observed in our study was significantly decreased in mothers of infants with CHD as compared with their controls, even after controlling for covariates. Similar result was presented in the study performed by Bahado-Singh et al.^29^. Through metabolomic analyses performed in pregnant women between 11 weeks’ and 14 weeks’ gestation, Bahado-Singh et al. found a significant reduction in sphingomyelin and acylcarnitine levels in pregnant women with CHD babies^29^. Existing evidence has proved that sphingomyelin contributed to the development of insulin resistance by modifying the insulin signaling pathway^30^. Based on this, we hypothesize that sphingomyelin may affect embryonic cardiovascular development via modification of the insulin signaling pathway; this need to be confirmed by further animal and human studies.

Through multi-omics analyses, a number of associations between the altered gut microbiota and plasma metabolites was found in this study. Basing on the Spearman correlation analyses, we found that the genera *Bifidobacterium* and *Streptococcus* were positively associated with six metabolites that were involved in lipid metabolism. The previous study reported that maternal lipid metabolism disturbance within 11 and 14 gestational weeks was associated with the development of CHD in offspring^29^. Therefore, it is possible that changes in the abundance of those genera, which were found to be associated with lipid metabolism in this study, may affect the fetal cardiovascular development by affecting maternal lipid metabolism. Furthermore, Sphingomyelin (d18:1/18:0), one of those six metabolites positively associated with *Bifidobacterium* and *Streptococcus*, was also enriched in sphingolipid signaling pathway that was proved to be related with insulin resistance. Considering the possible relationship between *Bifidobacterium* and insulin resistance as discussed earlier, we believe that *Bifidobacterium* may play an important role in the association of maternal gut microbiota with risk of CHD in offspring.

An important limitation, which must be noted and existed in our study, was the application of case–control study design; it may be uncapable to identify the real relationship between maternal gut microbiota and risk of CHD in offspring as well as the relevant mechanisms, given that the key development phase of CHD is the third to eighth weeks during pregnancy. Based on the available evidences on gut microbiota and its impact factors, the view that maternal gut microbiota after 1 year postpartum has back to the status prior to pregnancy to a certain extent is reasonable. Therefore, as a preliminary exploration of the association between maternal gut microbiota and risk of CHD in offspring, the present case–control study is valuable. In this study, efforts were made to minimize the influence of confounding factors on the association, including the application of a series of strict selection criteria to screen participants and controlling for covariates in the process of statistical analysis, which can help to support the reliability of our results.

A further limitation of the present study is the heterogeneity of the case population by lumping mothers of infants with all CHDs in analysis. Among those infants with CHD, 87 had more than one distinct CHDs (e.g., ventricular septal defect with mitral insufficiency). Therefore, we cannot assess the association for specific defects. Further studies concerning the association between maternal gut microbiota and specific defects can help to provide more information about the disease. In addition, this study is a hospital-based case–control study, the participants recruited from the hospital according to our strict selection criteria may be different from mothers of infants with CHD randomly chosen from a community. This limitation may introduce admission rate bias, which limits the generalizability of our findings.

Taken together, our findings show that mothers of infants with CHD have different gut microbiota composition and metabolic profiles as compared with mothers of infants without CHD, and represents an initial and critical step towards understanding the relationship between maternal gut microbiome and risk of CHD in offspring. Future studies are needed to address several interesting questions in this exciting filed. Firstly, it needs to be determined whether there is a causal link between any changes in maternal gut microbiota as well as its associated metabolites and the development of CHD in offspring. Considering that each subtype of CHD may not have the same pathological mechanism, analyses performed in specific defects like isolated ventricular septal defect will make more sense. If causality is determined, the identification of key functional bacteria taxa and related mechanisms is also needed; this may be beneficial for further research on the prevention of CHD. As we said at the beginning, a prospective preconception cohort study is the most effective way to address these questions. Taken into account the relatively low birth prevalence of CHD, and, thereby, high financial costs, it must be a long and arduous process.

In conclusion, 16S rRNA sequencing and metabolomics were combined in this study to explore the association between alterations in maternal gut microbiota and plasma metabolites and risk of CHD in offspring. The sequencing revealed that the gut microbiome composition was significantly different in mothers of infants with CHD as compared with their controls, whereas the results of metabolomics showed that many plasma metabolites involved in diverse metabolic pathways were significantly different between the two groups. In additional, correlation analysis identified a number of correlations between altered bacteria genera and plasma metabolites. Overall, these data suggesting that perturbations of maternal gut microbiota and plasma metabolites may be associated with risk of CHD in offspring, and co-variation between microbiota and metabolites may play a part in the linkage between gut microbiota and risk of CHD in offspring. These findings may provide new insights for revealing the novel potential etiology of CHD, understanding the role of maternal gut microbiota in CHD in offspring, and modulating gut microbiota as a therapeutic target.

## Methods

### Ethics statement

The study was approved by the Ethics Committee of Xiangya School of Public Health Central South University (No. XYGW-2018-36) and performed according to the Declaration of Helsinki. The design and purpose of the current study were clearly described in the research protocol; the protocol was registered at the Chinese Clinical Trial Registry with registration number ChiCTR1800018492 and is available at http://www.chictr.org.cn/listbycreater.aspx. All participants provided written informed consent before completing an enrollment questionnaire as well as providing biological samples.

### Study design and subjects

A hospital-based case–control design was used. Cases and controls were recruited from mothers of children who were 1 year of age or older and presented in Hunan Children’s Hospital during March 2018 and February 2019 in Hunan, China.

In this study, CHD was defined as a severe structural abnormality in the heart or intrathoracic great vessels that are possibly or actually of functional significance^31^. All CHD cases were diagnosed by pediatric cardiologists through routine examination, heart auscultation, and echocardiography, with some diagnoses further confirmed through cardiac surgery or catheterization during the period. The confirmed cases were classified according to the International Classification of Diseases-10 (ICD-10). Mothers whose children were diagnosed with CHD without other congenital diseases were chosen as cases, and mothers (from the same hospital) whose children had neither CHD nor other congenital disease were chosen as controls. Any participants who met any of the following criteria were excluded from this study: (1) multiple pregnancies; (2) unable to cooperate with the investigation due to mental illness or extreme emotional instability; (3) woman whose child had any congenital disease other than CHD, or tumour; (4) any congenital diseases or family history of congenital disease; (5) parous woman who was lactating; (6) any suspected or confirmed immunosuppression or immunodeficiency (acquired or primary) including human immunodeficiency virus infection; (6) any history of gastrointestinal tumor or currently suffering from any cancer/tumor; (7) any gastrointestinal diseases or non-gastrointestinal acute disease at the time of enrollment; (8) oral or intravenous antibiotics within the last 6 weeks; (9) use of insulin or corticosteroids at the time of enrollment; (10) long-term treatment with non-steroidal anti-inflammatory drugs, such as aspirin; (11) commercial probiotics or prebiotics consumed within the last 2 weeks; (12) parous woman who did not provide blood or stool sample, or complete the questionnaire.

### Sample and information collection

Overall, the study comprised analyses of microbial DNA isolated from fecal samples and metabolites in plasma samples of 196 parous women: 101 mothers of infants with CHD (cases) and 95 mothers of infants with CHD (controls). Peripheral venous blood was collected in the morning the day after enrollment and placed immediately after collection at 4 °C. Each woman was given a fecal sampler and provided detailed instructions for sample collection. Fecal samples freshly collected from each woman were placed immediately at − 18 °C. All samples were transported to the laboratory within 12 h and stored at − 80 °C until processing. In addition, information related to maternal socio-demographic characteristics, diet, lifestyles, disease and health conditions, medication and dietary supplement use were captured in a detailed questionnaire completed by each participant upon enrollment and further confirmed by consulting their Maternal and Child Health Manual and medical records. Information on the baby’s gender, age, and final diagnosis was collected by medical records at Hunan Children’s Hospital.

### DNA extraction, sequencing, and data processing

Bacterial DNA was extracted from a total of 196 frozen fecal samples using the standardization CTAB (cetyl trimethylammonium bromide) method as previously described^32^. Isolated bacterial DNA was then used as a template for the amplification of the V4 region of 16S rRNA gene using the 515F/806R primer for PCR^33^. A sequencing library of the V4 region was generated using TruSeq DNA PCR-Free Sample Preparation Kit (Illumina, USA) following manufacturer's recommendations. For PCR products, Illumina-based sequencing was carried out on an Illumina HiSeq2500 platform (Illumina Inc., San Diego, CA, USA) using the pair-end 250 bp protocol.

De-multiplexing 16S rRNA gene sequences and quality control were performed using the open-source bioinformatics pipeline Quantitative Insights into Microbial Ecology (QIIME) version 1.9.1^34^. Filtered sequences were clustered into operational taxonomic units (OTUs) based on 97% identity using UPARSE version 7.0.1001^35^. Representative sequences for each OTU were subjected to the SILVA132 database using the Mother software package to determine the phylogeny with a bootstrap cut-off of 80%, and built into a phylogenetic tree using MUSCLE version 3.8.3^36,37^. The sequences of all samples were downsized to 44,234 to match the difference in sequencing depth.

Alpha- and beta-diversity analyses were performed using QIIME version 1.9.1^38^, basing on a normalized OTU table. Alpha-diversity, within sample diversity, was calculated using Shannon index, Chao1 index, and the observed species^39^. Statistical significance of alpha-diversity between the case and control groups was analyzed with the linear regression analysis which was performed in SPSS 24.0 (IBM, New York, USA). Beta-diversity, between sample diversity, was calculated with unweighted and weighted UniFrac distance matrices and visualized using the score plot of the principal coordinate analysis (PCoA)^40,41^. Multifactorial permutational analysis of variance (PERMANOVA) (*Adonis* function, vegan package, R) of the UniFrac distance was used to test the association of infant’s CHD status with the microbiome beta-diversity while adjusting for covariates and multiple hypothesis testing. The line discriminant analysis (LDA) of effect size (LEfSe) was used to estimate the effect of the abundance of each microbiota taxa on the difference effect, and to identify microbiota taxa that have significant differences in their demarcation^42^. False discovery rate (FDR) was calculated according to Benjamini-Hochberg, FDR-corrected p-value was denoted as Q_FDR_ and was used for multiple test corrections. Taxa that showed an LDA score > 2 at a Q_FDR_ < 0.05 were selected for further analysis. Multivariate Association with Linear Models (MaAslin) was applied for association testing of the metadata versus the abundance of the differential bacterial taxa identified in the LEfSe analysis, to deconfound the effects of covariates. Both the LEfSe and MaAslin were accessed online from http://huttenhower.sph.harvard.edu/galaxy with default parameters. For linear regression analysis, PERMANOVA, and MaAslin, age, BMI, ethnicity, residence, cigarette smoking, alcohol consumption, negative life events, and dietary intake were used as covariates.

### Untargeted metabolomics analysis and data processing

Ultra-high-performance liquid chromatography-mass spectrometry (UPLC-MS)-based metabolic profiling of plasma samples was performed on an Agilent 1290 Infinity UPLC system (Agilent, Santa-Clara, California, USA) coupled with a Triple TOF 5600 Micromass system (AB SCIEX, Framingham, MA, USA) in both positive and negative ionization modes.

Plasma samples stored at − 80 °C were gradually thawed at 4 °C and 100 µL aliquots were mixed with 400 μL of cold methanol/acetonitrile (1:1, v/v). The mixture was vortexed for 1 min, placed at − 20 °C for 10 min, and centrifuged for 20 min (14,000 *g*, 4 °C). The supernatant was dried in a vacuum centrifuge. For the MC analysis, the samples were re-dissolved in 100 μL acetonitrile/water (1:1, v/v)^43^. Quality control (QC) samples were prepared by mixing all the samples equally as a pooled sample, and then analyzed together with the other samples. Regular intervals to insert a QC sample (every 20 samples) throughout the analytical process provide a set of data from which stability and repeatability can be assessed.

Chromatographic analysis was performed using an Agilent UPLC system. Chromatographic separation was run on ACQUITY BEH Amide 1.7 μm (2.1×100 mm) columns for both negative and positive models. The injection volume was 2.0 μL and the column temperature was set at 25 °C. The following gradients were used for separation: 95% B over 0–1 min, 95–65% B over 1–14 min, 40% B for 2 min, 40–95% B over 18–18.1 min, and 18.1–23 min holding at 95% at a flow rate of 0.3 mL/min, where B is acetonitrile and A is an aqueous solution containing ammonium acetate (25 mL) and ammonia (25 mL).

After separation by UPLC, mass spectrometry was performed using a Triple TOF 5600 mass spectrometer equipped with an electrospray ionization (ESI) source operating in either negative or positive ion mode. ESI source conditions were set as follows: nebulizer gas (ion source gas 1), 60 psi; auxiliary heating gas (ion source gas 2), 60 psi; curtain gas, 30 psi; source temperature, 600 °C; ion spray voltage floating, ± 5500 V. The MS/MS spectra were detected using information-based acquisition (IDA) which was an artificial intelligence-based product ion scan mode. The parameters for IDA were set as following: decluttering potential, ± 60 V; collision energy, 35 ± 15 eV; exclude isotopes within 4 Da, candidate ions monitor per cycle: 6.

The UPLC-Q-TOF/MS raw data were converted to mzXML files using Proteo Wizard tool and then processed using XCMS software for peak alignment to obtain a peak list containing the *m/z*, retention time, and peak area of each sample. For peak picking, the following settings were used: centWave *m/z* = 25 ppm, peakwidth = c (10, 60), prefilter = c (10, 100). By using *m/z* and retention time pairs as identifiers for each ion, we obtained the ion intensity of each peak and generated a matrix containing sample names, arbitrarily specified peak index (retention time-*m/z* pairs), and ion intensities. After ion features were extracted, only the variables having more than 2/3 of the nonzero measurement values in all samples were kept for subsequent analysis. After being normalized based on peak areas, the positive and negative data were combined into a data set and imported into SIMCA-P 14.1 software (Umetrics, Umea, Sweden) for multivariate statistical analysis.

A supervised orthogonal partial least squares-discrimination analysis (OPLS-DA) model was used to show statistical differences and identify differentially expressed metabolites in mothers of infants with CHD relative to the controls, and this model was validated by a permutation test with 200 iterations^44^. The variable importance for the projection (VIP) value of each variable in the OPLS-DA model was calculated to indicate its contribution to the classification. The differentially expressed metabolites were recognized by using two-tailed Student’s t-test and multivariable linear regression. The model adjusted for potential confounders including age, BMI, ethnicity, residence, cigarette smoking, alcohol consumption, negative life events, and dietary intake. P-value was adjusted for multiple comparisons with the FDR algorithm according to Benjamini-Hochberg. Metabolite structural identification was performed by accurate matching of mass number (< 25 ppm) and matching of second-stage spectra with the laboratory self-built database. All metabolites were identified to level 1 according to the Metabolomics Standards Initiative (MSI)^45^. Based on the differentially expressed metabolites, Kyoto Encyclopedia of Genes and Genomes (KEGG) pathway analysis (http://www.genome.jp/kegg/) was conducted to investigate the metabolomic pathways associated with the disease status of the offspring^46^. The enrichment level of each metabolomic pathway was calculated by Fisher's exact test, and a p-value < 0.05 was regarded as statistically significant.

### Spearman multi-omic correlation analysis

Spearman correlation between the relative abundance of genera and the level of plasma metabolites was analyzed using R software. We only performed the correlation in those genera and metabolites which were found to be statistically significant between groups while controlling for potential confounders as mentioned above (p < 0.05), and then, presented the results by heatmap (for all correlations) and network plot (for correlations with absolute value above 0.3).

### Statistical analyses

The statistical analysis was performed in SPSS 24.0 (IBM, New York, USA). The results were presented as a mean value (standard error, SD) for continuous variables, or as a proportion for categorical variables. The t-test was used for continuous variables and the chi-square test for categorical variables to identify significant differences between the case and control groups. A value of p < 0.05 was considered as significant.

### Definitions

The definitions of important items including gut microbiota, metabolomics, and 16 S rRNA gene sequencing were shown in Supplementary File 1.

## Supplementary Information

Supplementary Information

## Data Availability

All data generated or analyzed during this study are included in this published article and its supplementary information files.
