# Supplementary material for: Association of maternal gut microbiota and plasma metabolism with congenital heart disease in offspring: a multi-omic analysis
Source: Sci Rep. 2021 Mar 5;11:5339. doi: 10.1038/s41598-021-84901-7 (PMC7935922; doi:10.1038/s41598-021-84901-7)
Supplement: Supplementary file 1 — Supplementary Information [file 41598_2021_84901_MOESM1_ESM.doc]

**Title page**

**Title:** Association of maternal gut microbiota and plasma metabolism with congenital heart disease in offspring: a multi-omic analysis

**Author names and affiliations:**

Tingting Wang, PHD,^1,2^ Lizhang Chen, PHD,^1,3^ Peng Huang, MD,^4^ Tubao Yang, PHD,^1^ Senmao Zhang, MPH,^1^ Lijuan Zhao, MPH,^1^ Letao Chen, MPH,^1^ Ziwei Ye, MPH,^1^ Liu Luo, MPH,^1^ Jiabi Qin, MD, PHD,^1,2,5*^

^1^Department of Epidemiology and Health Statistics, Xiangya School of Public Health, Central South University, Hunan, China

^2^National Health Commission Key Laboratory for Birth Defect Research and Prevention, Hunan Provincial Maternal and Child Health Care Hospital, Hunan, China

^3^Hunan Provincial Key Laboratory of Clinical Epidemiology, Changsha, Hunan, China

^4^Department of Thoracic Cardiac Surgery, Hunan Children's Hospital, Hunan, China

^5^Guangdong Cardiovascular Institute, Guangdong Provincial People's Hospital, Guangdong Academy of Medical Sciences, Guangzhou, Guangdong, China

***Corresponding author**

Jiabi Qin, Department of Epidemiology and Health Statistics, Xiangya School of Public Health, Central South University, NO. 238 Shang Ma Yuan Ling Xiang Xiangya Road, Kaifu District, Changsha, Hunan 410078, China. Fax: 0731-84805414, Telephone: 0731-84805414, E-mail: [qinjiabi123@163.com](mailto:qinjiabi123@163.com).

**Supplementary Information**

**Supplementary Figure 1** Alpha-diversity rarefaction curves.

**Supplementary Figure 2** PCoA based on weighted UniFrac distance.

**Supplementary Figure 3** Loading plots generated by OPLS-DA analysis.

**Supplementary Figure 4** Violin plot showing the abundance of 58 differentially expressed plasma metabolites in mothers of infants with CHD and the control group.

**Supplementary Figure 5** KEGG pathway analysis of the differential metabolite components in mothers of infants with CHD and the control group.

**Supplementary Figure 6** Heatmap summarizing the correlation between the abundant bacterial genera and differentially expressed metabolites.

**Supplementary Table 1** Characteristics of mothers of infants with CHD and the control group.

**Supplementary Table 2** Linear regression analyses of alpha diversity (Shannon index, Chao1 index, and observed species).

**Supplementary Table 3** PERMANOVA analysis based on the unweighted UniFrac distance.

**Supplementary Table 4** Differentially abundant bacterial taxa at the genus level identified by LEfSe and MaAsLin analyses.

**Supplementary Table 5** Differentially expressed plasma metabolites identified by univariate and multivariate analyses.

**Supplementary File 1** Definitions.


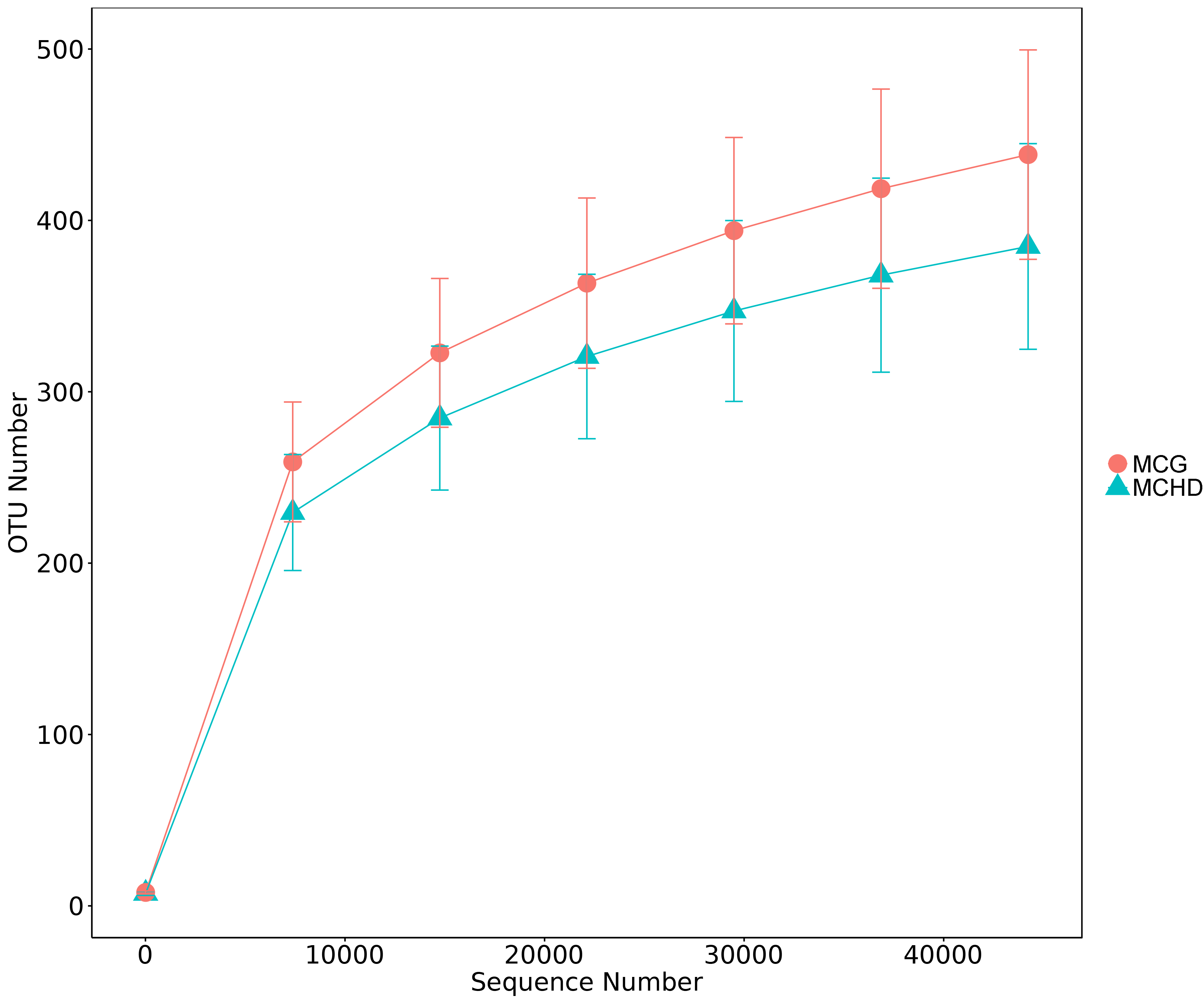


**Supplementary Figure 1 Alpha-diversity rarefaction curves.** X-axis reports the number of reads per sample and Y-axis shows the number of OTUs. OTUs, operational taxonomic units


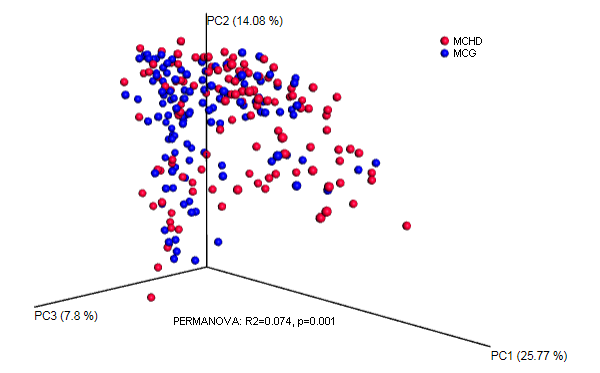


**Supplementary Figure 2 PCoA based on weighted UniFrac distance.** The result showed that the overall gut microbiota composition was different between mothers of infants with CHD and controls (R^2^ = 0.074, p = 0.001). CHD, congenital heart disease; PCoA, principal coordinates analysis.





**Supplementary Figure 3 Loading plots generated by OPLS-DA analysis.**

**A**





**B**





**C**



**Supplementary Figure 4 Violin plot showing the abundance of 58 differentially expressed plasma metabolites in mothers of infants with CHD and the control group.** The orange violin plot represents mothers of infants with CHD, and the green represents mothers of infants without CHD. CHD, congenital heart disease.


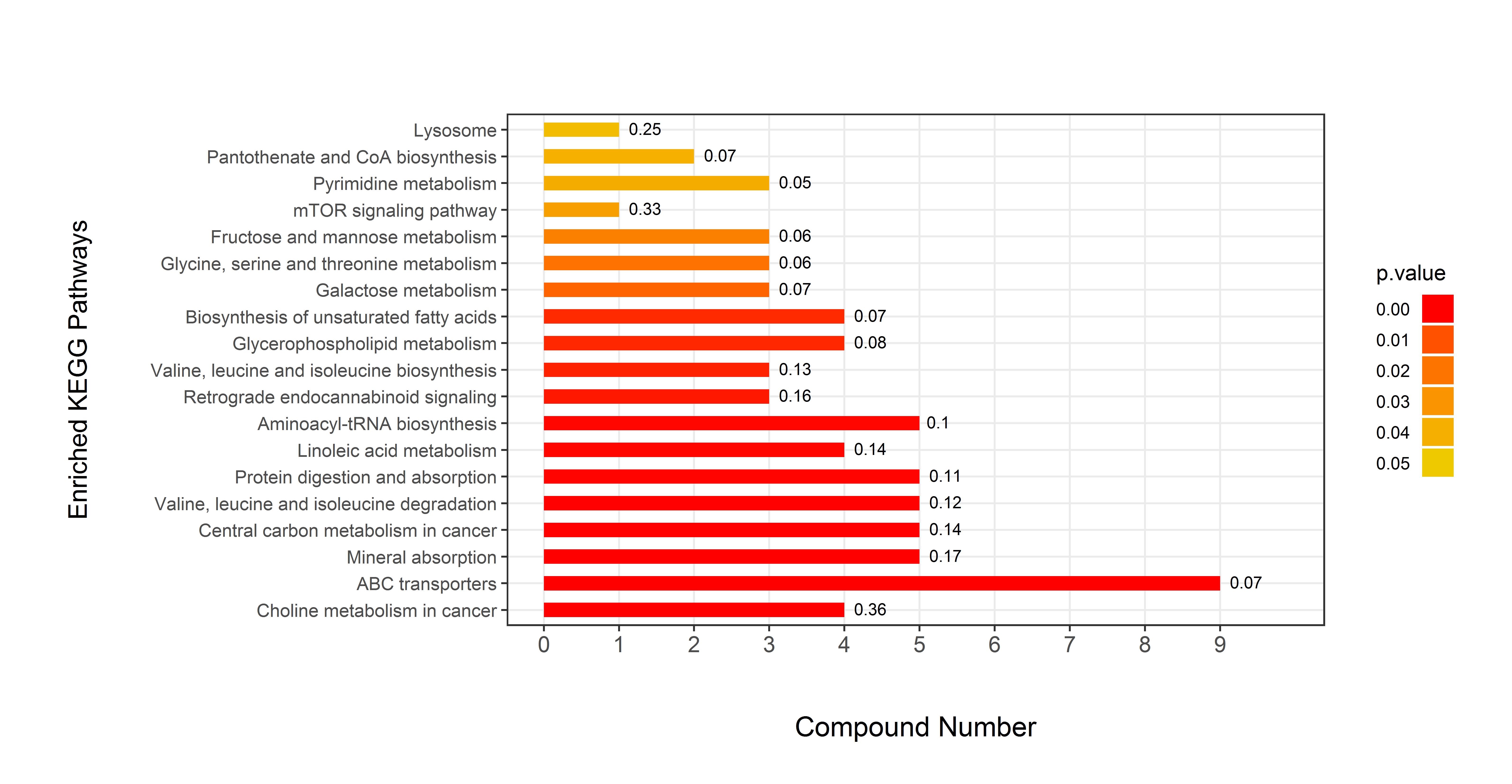


**Supplementary Figure 5 KEGG ^1^ pathway analysis of the differential metabolite components in mothers of infants with CHD and the control group.** KEGG, Kyoto Encyclopedia of Genes and Genomes.

1. Kanehisa, M., Goto, S. KEGG: Kyoto Encyclopedia of Genes and Genomes. *Nucleic Acids Res* **28**, 27-30 (2000).


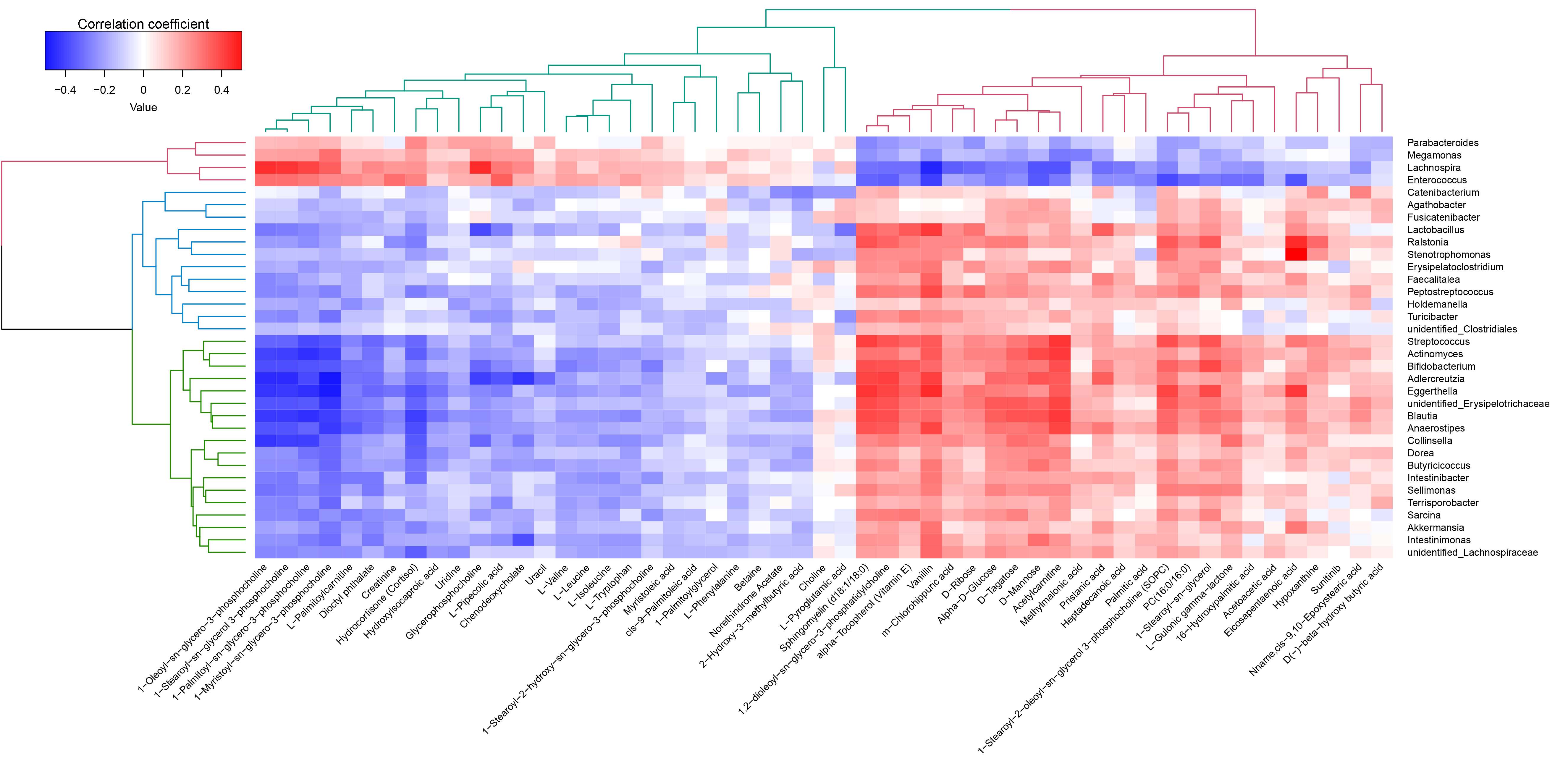


**Supplementary Figure 6 Heatmap summarizing the correlation between the abundant bacterial genera and differentially expressed metabolites.**

**Supplementary Table 1 Characteristics of participants and their infants.**

| Characteristic | Mothers of infants with CHD (N = 101), n (%) | Mothers of infants without CHD (N = 95), n (%) | p-value |
| --- | --- | --- | --- |
| **Mean (SD) age** (years) | 30.82 (5.97) | 31.60 (4.91) | 0.323 |
| **Mean (SD) BMI** (kg/m^2^) | 21.73 (3.83) | 21.66 (3.08) | 0.879 |
| **Ethnicity** |  |  | **0.030** |
| Han nationality | 85 (84.2) | 90 (94.7) |  |
| Miao nationality | 9 (8.9) | 4 (4.2) |  |
| Others | 7 (6.9) | 1 (1.1) |  |
| **Residence** |  |  | 0.129 |
| Rural | 73 (72.3) | 59 (62.1) |  |
| Urban | 28 (27.7) | 36 (37.9) |  |
| **History of diabetes** |  |  | - |
| No | 100 (99.0) | 95 (100) |  |
| Yes | 1 (1.0) | 0 (0.0) |  |
| **Cigarette smoking** |  |  | 1.000 |
| No | 100 (99.0) | 94 (98.9) |  |
| Yes | 1 (1.0) | 1 (1.1) |  |
| **Alcohol consumption** |  |  | 0.516 |
| No | 94 (93.1) | 86 (90.5) |  |
| Yes | 7 (6.9) | 9 (9.5) |  |
| **Negative life events^*^** |  |  | 0.884 |
| No | 93 (92.1) | 88 (92.6) |  |
| Yes | 8 (7.9) | 7 (7.4) |  |
| **Dietary intake** |  |  |  |
| Cereal products (rice, steamed buns, noodles, and so on) |  |  | - |
| ≥ 6 times/week | 101 (100) | 95 (100) |  |
| 3-5 times/week | 0 (0) | 0 (0) |  |
| 1-2 times/week | 0 (0) | 0 (0) |  |
| < 1 times/week | 0 (0) | 0 (0) |  |
| Tubers products (sweet potato, potato, taros, and so on) |  |  | 0.445 |
| ≥ 6 times/week | 34 (33.7) | 39 (41.1) |  |
| 3-5 times/week | 57 (56.4) | 50 (52.6) |  |
| 1-2 times/week | 10 (9.9) | 6 (6.3) |  |
| < 1 times/week | 0 (0.0) | 0 (0.0) |  |
| Vegetables (leafy greens, green pepper, carrot, and so on) |  |  | 0.995 |
| ≥ 6 times/week | 96 (95.0) | 90 (94.7) |  |
| 3-5 times/week | 3 (3.0) | 3 (3.2) |  |
| 1-2 times/week | 2 (2.0) | 2 (2.1) |  |
| < 1 times/week | 0 (0.0) | 0 (0.0) |  |
| Fruits |  |  | 0.110 |
| ≥ 6 times/week | 74 (73.3) | 75 (78.9) |  |
| 3-5 times/week | 13 (12.9) | 16 (16.8) |  |
| 1-2 times/week | 11 (10.9) | 3 (3.2) |  |
| < 1 times/week | 3 (3.0) | 1 (1.1) |  |
| Livestock and poultry meat (pork, beef, chicken, and so on) |  |  | 0.540 |
| ≥ 6 times/week | 81 (80.2) | 77 (81.1) |  |
| 3-5 times/week | 10 (9.9) | 13 (13.7) |  |
| 1-2 times/week | 7 (6.9) | 3 (3.2) |  |
| < 1 times/week | 3 (3.0) | 2 (2.1) |  |
| Water products (fish, shrimp, and so on) |  |  | < 0.001 |
| ≥ 6 times/week | 13 (12.9) | 36 (37.9) |  |
| 3-5 times/week | 19 (18.8) | 24 (25.3) |  |
| 1-2 times/week | 51 (50.5) | 30 (31.6) |  |
| < 1 times/week | 18 (17.8) | 5 (5.3) |  |
| Eggs (fresh eggs, preserved eggs, and salted eggs) |  |  | 0.005 |
| ≥ 6 times/week | 44 (43.6) | 58 (61.1) |  |
| 3-5 times/week | 20 (19.8) | 23 (24.2) |  |
| 1-2 times/week | 30 (29.7) | 12 (12.6) |  |
| < 1 times/week | 7 (6.9) | 2 (2.1) |  |
| Milk/dairy products (milk, liquid milk, cheese, and so on) |  |  | 0.125 |
| ≥ 6 times/week | 39 (38.6) | 31 (32.6) |  |
| 3-5 times/week | 13 (12.9) | 21 (22.1) |  |
| 1-2 times/week | 20 (19.8) | 25 (26.3) |  |
| < 1 times/week | 29 (28.7) | 18 (18.9) |  |
| Beans/bean products (soybean, soya-bean milk, tofu, and so on) |  |  | 0.004 |
| ≥ 6 times/week | 17 (16.8) | 28 (29.5) |  |
| 3-5 times/week | 18 (17.8) | 28 (29.5) |  |
| 1-2 times/week | 46 (45.5) | 22 (23.2) |  |
| < 1 times/week | 20 (19.8) | 17 (17.9) |  |
| Smoked food |  |  | 0.421 |
| ≥ 6 times/week | 2 (2.0) | 0 (0.0) |  |
| 3-5 times/week | 4 (4.0) | 4 (4.2) |  |
| 1-2 times/week | 37 (36.6) | 33 (34.7) |  |
| < 1 times/week | 58 (57.4) | 58 (61.1) |  |
| Grilled food |  |  | 0.387 |
| ≥ 6 times/week | 1 (1.0) | 0 (0.0) |  |
| 3-5 times/week | 1 (1.0) | 1 (1.1) |  |
| 1-2 times/week | 14 (13.9) | 20 (21.1) |  |
| < 1 times/week | 85 (84.2) | 74 (77.9) |  |
| Fried food |  |  | 0.053 |
| ≥ 6 times/week | 1 (1.0) | 0 (0.0) |  |
| 3-5 times/week | 2 (2.0) | 1 (1.1) |  |
| 1-2 times/week | 19 (18.8) | 33 (34.7) |  |
| < 1 times/week | 79 (78.2) | 61 (64.2) |  |
| **Mean (SD) age of infants** (years) | 1.84 (0.57) | 1.95 (0.57) | 0.186 |
| **Gender of infants** |  |  | **0.026** |
| Male | 50 (49.5) | 62 (65.3) |  |
| Female | 51 (50.5) | 33 (34.7) |  |
| **Mean (SD) gestational age of infants** (weeks) | 39.07(1.50) | 39.3 (1.14) | 0.256 |
| **Type of CHD^#^** |  |  |  |
| Arial septal defect | 26 (25.7%) | - | - |
| Vntricular septal defect | 65 (64.4%) | - | - |
| Arioventricular septal defect | 8 (7.9%) | - | - |
| Ptent ductus arteriosus | 24 (23.8%) | - | - |
| Ttralogy of fallot | 6 (5.9%) | - | - |

*a negative life event is something that can make the person feel pain and distress about it, and as a result, may cause the person to have negative emotions such as anxiety and depression. For example, divorce, death of relatives, and etc.

#some cases have been diagnosed with multiple subtypes of CHD. Thus, the sum of the various subtypes was not equal to 101.

BMI, body mass index; MCHD, mothers of infants with CHD; MCG, mothers of infants without CHD; SD, standard deviation.

**Supplementary Table 2 Linear regression analyses of alpha diversity (Shannon index, Chao1 index, and observed species).**

**Shannon index**

| Variables | Unadjusted | | | Adjusted | | |
| --- | --- | --- | --- | --- | --- | --- |
|  | Standardized coefficient | t | p-value | Standardized coefficient | t | p-value |
| **CHD** | -0.317 | -4.661 | **< 0.001** | -0.29 | -4.124 | **< 0.001** |
| **Age** | -0.022 | -0.306 | 0.76 | - | - | - |
| **BMI** | -0.247 | -3.550 | **< 0.001** | -0.221 | -3.315 | **0.001** |
| **Ethnicity** (Han nationality) | -0.110 | -1.546 | 0.124 | - | - | - |
| **Residence** (rural) | 0.113 | 1.589 | 0.114 | - | - | - |
| **Cigarette_smoking** (yes) | -0.032 | -0.448 | 0.655 | - | - | - |
| **Alcohol_consumption** (yes) | -0.117 | -1.647 | 0.101 | - | - | - |
| **Negative_life_events** (yes) | -0.156 | -2.198 | **0.029** | -0.139 | -2.115 | **0.036** |
| **Dietary intake** |  |  |  |  |  |  |
| Cereal products | - | - | - | - | - | - |
| Tubers products | 0.133 | 1.866 | 0.064 | - | - | - |
| Vegetables | 0.112 | 1.572 | 0.118 | - | - | - |
| Fruits | 0.162 | 2.281 | **0.024** | 0.086 | 1.246 | 0.214 |
| Livestock and poultry meat | 0.104 | 1.459 | 0.146 | - | - | - |
| Water products | 0.171 | 2.407 | **0.017** | 0.028 | 0.379 | 0.705 |
| Eggs | -0.006 | -0.088 | 0.93 | - | - | - |
| Milk | 0.111 | 1.550 | 0.123 | - | - | - |
| Beans/bean products | 0.109 | 1.523 | 0.129 | - | - | - |
| Smoked food | -0.043 | -0.598 | 0.55 | - | - | - |
| Grilled food | 0.025 | 0.354 | 0.724 | - | - | - |
| Fried food | 0.086 | 1.205 | 0.230 | - | - | - |

For the multivariate analyses, the variables including age, BMI, ethnicity, residence, alcohol consumption, negative life events, and dietary intake that showed an effect in the univariate analyses with a p-value < 0.05 were included as covariates. Significant p-values (p < 0.05) are shown in bold.

BMI, body mass index; CHD, congenital heart disease.

**Chao1 index**

| Variables | Unadjusted | | | Adjusted | | |
| --- | --- | --- | --- | --- | --- | --- |
|  | Standardized coefficient | t | p-value | Standardized coefficient | t | p-value |
| **CHD** | -0.400 | -6.086 | **< 0.001** | -0.376 | -5.388 | **< 0.001** |
| **Age** | 0.051 | 0.713 | 0.477 | - | - | - |
| **BMI** | -0.174 | -2.454 | **0.015** | -0.142 | -2.159 | **0.032** |
| **Ethnicity** (Han nationality) | -0.091 | -1.276 | 0.204 | - | - | - |
| **Residence** (rural) | 0.104 | 1.452 | 0.148 | - | - | - |
| **Cigarette_smoking** (yes) | -0.007 | -0.096 | 0.924 | - | - | - |
| **Alcohol_consumption** (yes) | 0.009 | 0.131 | 0.896 | - | - | - |
| **Negative_life_events** (yes) | -0.085 | -1.189 | 0.236 | 0.135 | 1.948 | 0.053 |
| **Dietary intake** |  |  |  |  |  |  |
| Cereal products | - | - | - | - | - | - |
| Tubers products | 0.150 | 2.110 | 0.036 | - | - | - |
| Vegetables | 0.063 | 0.873 | 0.384 | - | - | - |
| Fruits | 0.212 | 3.017 | **0.003** | 0.019 | 0.265 | 0.791 |
| Livestock and poultry meat | -0.042 | 0.590 | 0.556 | - | - | - |
| Water products | 0.202 | 2.861 | **0.005** | 0.016 | 0.236 | 0.813 |
| Eggs | 0.109 | 1.534 | 0.127 | - | - | - |
| Milk | 0.059 | 0.826 | 0.410 | - | - | - |
| Beans/bean products | 0.147 | 2.075 | **0.039** | - | - | - |
| Smoked food | -0.094 | -1.314 | 0.190 | - | - | - |
| Grilled food | 0.021 | 0.299 | 0.765 | - | - | - |
| Fried food | 0.036 | 0.508 | 0.612 | - | - | - |

For the multivariate analyses, the variables including age, BMI, ethnicity, residence, alcohol consumption, negative life events, and dietary intake that showed an effect in the univariate analyses with a p-value < 0.05 were included as covariates. Significant p-values (p < 0.05) are shown in bold.

BMI, body mass index; CHD, congenital heart disease.

**Observed_species**

| Variables | Univariate | | | Multivariate | | |
| --- | --- | --- | --- | --- | --- | --- |
|  | Standardized coefficient | t | p-value | Standardized coefficient | t | p-value |
| **CHD** | -0.393 | -5.947 | **< 0.001** | -0.371 | -5.349 | **< 0.001** |
| **Age** | 0.046 | 0.640 | 0.523 | - | - | - |
| **BMI** | -0.188 | -2.673 | **0.008** | -0.161 | -2.46 | **0.015** |
| **Ethnicity** (Han nationality) | -0.080 | -1.113 | 0.267 | - | - | - |
| **Residence** (rural) | 0.122 | 1.170 | 0.089 | - | - | - |
| **Cigarette_smoking** (yes) | -0.013 | 0.186 | 0.852 | - | - | - |
| **Alcohol_consumption** (yes) | -0.010 | 0.132 | 0.895 | - | - | - |
| **Negative_life_events** (yes) | -0.108 | 1.509 | 0.133 | - | - | - |
| **Dietary intake** |  |  |  |  |  |  |
| Cereal products | - | - | - | - | - | - |
| Tubers products | 0.139 | 1.949 | 0.053 | - | - | - |
| Vegetables | 0.065 | 0.908 | 0.365 | - | - | - |
| Fruits | 0.201 | 2.852 | **0.005** | 0.125 | 1.84 | 0.067 |
| Livestock and poultry meat | -0.035 | -0.489 | 0.625 | - | - | - |
| Water products | 0.192 | 2.714 | **0.007** | 0.017 | 0.234 | 0.815 |
| Eggs | 0.104 | 1.458 | 0.146 | - | - | - |
| Milk | 0.082 | 1.141 | 0.255 | - | - | - |
| Beans/bean products | 0.139 | 1.960 | 0.051 | - | - | - |
| Smoked food | -0.080 | -1.121 | 0.264 | - | - | - |
| Grilled food | 0.014 | 0.193 | 0.847 | - | - | - |
| Fried food | 0.049 | 0.687 | 0.493 | - | - | - |

For the multivariate analyses, the variables including age, BMI, ethnicity, residence, alcohol consumption, negative life events, and dietary intake that showed an effect in the univariate analyses with a p-value < 0.05 were included as covariates. Significant p-values (p < 0.05) are shown in bold.

BMI, body mass index; CHD, congenital heart disease.

**Supplementary Table 3 PERMANOVA analysis based on the unweighted UniFrac distance.**

| Variables | Univariate | | | Multivariate | | |
| --- | --- | --- | --- | --- | --- | --- |
|  | F.Model | R2 | p-value | F.Model | R2 | p-value |
| **Group** | 32.585 | 0.144 | **0.001** | 32.516 | 0.14381 | **0.001** |
| **Age** | 1.182 | 0.006 | 0.253 | - | - | - |
| **BMI** | 1.434 | 0.007 | 0.166 | - | - | - |
| **Ethnicity** | 0.960 | 0.005 | 0.358 | - | - | - |
| **Residence** | 0.972 | 0.005 | 0.400 | - | - | - |
| **Cigarette_smoking** | 0.410 | 0.002 | 0.956 | - | - | - |
| **Alcohol_consumption** | 2.000 | 0.010 | 0.074 | - | - | - |
| **Negative_life_events** | 0.555 | 0.003 | 0.839 | - | - | - |
| **Dietary intake** |  |  |  |  |  |  |
| Cereal products | - | - | - | - | - | - |
| Tubers products | 0.802 | 0.004 | 0.503 | - | - | - |
| Vegetables | 1.160 | 0.006 | 0.271 | - | - | - |
| Fruits | 0.763 | 0.004 | 0.549 | - | - | - |
| Livestock and poultry meat | 0.556 | 0.003 | 0.789 | - | - | - |
| Water products | 3.885 | 0.020 | **0.013** | 0.589 | 0.0026 | 0.742 |
| Eggs | 1.694 | 0.009 | 0.108 | - | - | - |
| Milk | 0.778 | 0.004 | 0.533 | - | - | - |
| Beans/bean products | 1.488 | 0.008 | 0.167 | - | - | - |
| Smoked food | 0.877 | 0.005 | 0.432 | - | - | - |
| Grilled food | 0.106 | 0.005 | 0.273 | - | - | - |
| Fried food | 1.819 | 0.009 | 0.105 | - | - | - |

For the multivariate analyses, the variables including age, BMI, ethnicity, residence, alcohol consumption, negative life events, and dietary intake that showed an effect in the univariate analyses with a p-value < 0.05 were included as covariates. Significant p-values (p < 0.05) are shown in bold.

BMI, body mass index.

**Supplementary Table 4 Differentially abundant bacterial taxa at the genus level identified by LEfSe and MaAsLin analyses.**

| Genus | Median abundance | | LEfSe | | | MaAsLin | | |
| --- | --- | --- | --- | --- | --- | --- | --- | --- |
|  |  |  | Enriched group | LDA | Q-FDR | Coefficient | p-value | Q-FDR |
| Blautia | 2332 | 6573 | CG | 4.472 | < 0.001 | -0.167 | < 0.001 | 0.001 |
| unidentified_Lachnospiraceae | 2145 | 4315 | CG | 4.047 | < 0.001< 0.001 | -0.053 | < 0.001< 0.001 | < 0.001< 0.001 |
| Bifidobacterium | 161 | 655 | CG | 3.783 | < 0.001< 0.001 | -0.045 | < 0.001< 0.001 | < 0.001< 0.001 |
| Streptococcus | 128 | 453 | CG | 3.726 | < 0.001< 0.001 | -0.026 | < 0.001< 0.001 | < 0.001< 0.001 |
| unidentified_Erysipelotrichaceae | 125 | 506 | CG | 3.717 | < 0.001< 0.001 | -0.028 | < 0.001< 0.001 | < 0.001 |
| Anaerostipes | 180 | 756 | CG | 3.681 | < 0.001 | -0.049 | < 0.001 | < 0.001 |
| Collinsella | 64 | 289 | CG | 3.678 | < 0.001 | -0.026 | < 0.001 | < 0.001 |
| unidentified_Ruminococcaceae | 1196 | 1804 | CG | 3.594 | 0.001 | - | - | - |
| Fusicatenibacter | 540 | 893 | CG | 3.581 | 0.001 | -0.021 | < 0.001 | 0.012 |
| Subdoligranulum | 641 | 1280 | CG | 3.514 | < 0.001 | - | - | - |
| Dorea | 384 | 808 | CG | 3.395 | < 0.001 | -0.026 | < 0.001 | < 0.001 |
| Lactobacillus | 15 | 53 | CG | 3.175 | < 0.001 | -0.009 | < 0.001 | < 0.001 |
| unidentified_Clostridiales | 135 | 218 | CG | 3.077 | 0.004 | -0.014 | < 0.001 | 0.012 |
| Holdemanella | 19 | 52 | CG | 2.939 | 0.011 | -0.008 | 0.001 | 0.018 |
| Butyricicoccus | 128 | 258 | CG | 2.936 | < 0.001 | -0.014 | < 0.001 | < 0.001 |
| Catenisphaera | 0 | 0 | CG | 2.866 | 0.048 | - | - | - |
| Intestinibacter | 50 | 125 | CG | 2.758 | < 0.001 | -0.014 | < 0.001 | < 0.001 |
| Catenibacterium | 0 | 5 | CG | 2.710 | < 0.001 | -0.004 | < 0.001 | 0.001 |
| Sellimonas | 7 | 29 | CG | 2.685 | < 0.001 | -0.008 | < 0.001 | 0.006 |
| Akkermansia | 3 | 12 | CG | 2.657 | < 0.001 | -0.004 | < 0.001 | < 0.001 |
| Sarcina | 0 | 0 | CG | 2.657 | < 0.001 | -0.002 | < 0.001 | < 0.001 |
| Adlercreutzia | 5 | 25 | CG | 2.623 | < 0.001 | -0.008 | < 0.001 | < 0.001 |
| Eggerthella | 7 | 35 | CG | 2.497 | < 0.001 | -0.010 | < 0.001 | < 0.001 |
| Desulfotalea | 0 | 0 | CG | 2.482 | 0.040 | - | - | - |
| Pygmaiobacter | 0 | 0 | CG | 2.421 | 0.019 | - | - | - |
| Desulfovibrio | 7 | 23 | CG | 2.408 | 0.005 | - | - | - |
| Ralstonia | 4 | 13 | CG | 2.373 | < 0.001 | -0.004 | < 0.001 | 0.001 |
| Acidipila | 0 | 0 | CG | 2.361 | 0.024 | - | - | - |
| Pseudoalteromonas | 0 | 0 | CG | 2.353 | 0.019 | - | - | - |
| Barnesiella | 15 | 30 | CG | 2.350 | 0.017 | - | - | - |
| Pseudofulvimonas | 0 | 0 | CG | 2.337 | 0.040 | - | - | - |
| Scardovia | 0 | 0 | CG | 2.319 | 0.024 | - | - | - |
| Paraclostridium | 0 | 0 | CG | 2.315 | 0.031 | - | - | - |
| Faecalitalea | 6 | 18 | CG | 2.296 | < 0.001 | -0.005 | < 0.001 | 0.002 |
| Pediococcus | 0 | 0 | CG | 2.291 | 0.004 | - | - | - |
| Roseiarcus | 0 | 0 | CG | 2.283 | 0.040 | - | - | - |
| unidentified_Desulfarculaceae | 0 | 0 | CG | 2.267 | 0.048 | - | - | - |
| Agathobacter | 41 | 64 | CG | 2.259 | 0.002 | -0.005 | 0.001 | 0.020 |
| Intestinimonas | 26 | 49 | CG | 2.237 | < 0.001 | -0.006 | < 0.001 | < 0.001 |
| Oligella | 0 | 0 | CG | 2.227 | 0.019 | - | - | - |
| Exiguobacterium | 0 | 1 | CG | 2.225 | < 0.001 | - | - | - |
| Turicibacter | 23 | 47 | CG | 2.191 | 0.001 | -0.006 | 0.001 | 0.039 |
| Paracoccus | 0 | 0 | CG | 2.188 | 0.019 | - | - | - |
| Sphingopyxis | 0 | 0 | CG | 2.182 | 0.040 | - | - | - |
| Terrisporobacter | 13 | 36 | CG | 2.168 | < 0.001 | -0.008 | < 0.001 | < 0.001 |
| Peptostreptococcus | 0 | 2 | CG | 2.152 | < 0.001 | -0.002 | < 0.001 | < 0.001 |
| unidentified_Anaerolineaceae | 0 | 0 | CG | 2.143 | 0.040 | - | - | - |
| Erysipelatoclostridium | 6 | 20 | CG | 2.117 | < 0.001 | -0.005 | < 0.001 | 0.005 |
| Actinomyces | 5 | 16 | CG | 2.106 | < 0.001 | -0.005 | < 0.001 | < 0.001 |
| Spongiimonas | 0 | 0 | CG | 2.097 | 0.040 | - | - | - |
| Desulfobacca | 0 | 0 | CG | 2.079 | 0.039 | - | - | - |
| Cetobacterium | 0 | 0 | CG | 2.076 | 0.025 | - | - | - |
| Duganella | 0 | 0 | CG | 2.074 | 0.047 | - | - | - |
| Finegoldia | 0 | 0 | CG | 2.072 | 0.005 | - | - | - |
| Acidibacter | 0 | 0 | CG | 2.047 | 0.038 | - | - | - |
| Polymorphobacter | 0 | 0 | CG | 2.046 | 0.040 | - | - | - |
| Peptoniphilus | 0 | 0 | CG | 2.007 | 0.019 | - | - | - |
| Psychrobacter | 0 | 0 | CG | 2.006 | 0.031 | - | - | - |
| Allobaculum | 0 | 0 | CG | 2.002 | < 0.001 | - | - | - |
| Deinococcus | 0 | 0 | CG | 2.000 | 0.040 | - | - | - |
| Bacteroides | 8542 | 5412 | CHD | 4.580 | 0.003 | - | - | - |
| Faecalibacterium | 10997 | 8328 | CHD | 4.200 | 0.047 | - | - | - |
| Megamonas | 769 | 248 | CHD | 4.056 | < 0.001 | 0.039 | < 0.001 | 0.004 |
| Lachnospira | 297 | 146 | CHD | 3.444 | < 0.001 | 0.024 | < 0.001 | < 0.001 |
| Parabacteroides | 380 | 217 | CHD | 3.442 | < 0.001 | 0.019 | 0.001 | 0.020 |
| Enterococcus | 126 | 29 | CHD | 3.333 | < 0.001 | 0.024 | < 0.001 | < 0.001 |
| Stenotrophomonas | 1 | 15 | CHD | 3.290 | < 0.001 | 0.009 | < 0.001 | < 0.001 |
| Romboutsia | 510 | 791 | CHD | 3.270 | 0.019 | - | - | - |
| Lachnoclostridium | 602 | 454 | CHD | 3.250 | 0.003 | - | - | - |
| Parasutterella | 97 | 56 | CHD | 3.134 | 0.001 | - | - | - |
| Alloprevotella | 20 | 4 | CHD | 2.845 | 0.001 | - | - | - |
| Desulfurivibrio | 0 | 0 | CHD | 2.581 | 0.021 | - | - | - |
| Inhella | 0 | 0 | CHD | 2.551 | 0.033 | - | - | - |
| Steroidobacter | 0 | 0 | CHD | 2.543 | 0.021 | - | - | - |
| Phyllobacterium | 5 | 13 | CHD | 2.533 | 0.038 | - | - | - |
| Alcanivorax | 0 | 0 | CHD | 2.490 | 0.006 | - | - | - |
| Thermodesulforhabdus | 0 | 0 | CHD | 2.476 | 0.002 | - | - | - |
| Aquimonas | 0 | 0 | CHD | 2.327 | 0.001 | - | - | - |
| Flavonifractor | 55 | 42 | CHD | 2.269 | 0.024 | - | - | - |
| Acidaminococcus | 0 | 0 | CHD | 2.260 | 0.023 | - | - | - |
| Thioalbus | 0 | 0 | CHD | 2.206 | 0.033 | - | - | - |
| unidentified_Acidobacteria | 0 | 0 | CHD | 2.204 | 0.026 | - | - | - |
| Sutterella | 15 | 10 | CHD | 2.192 | 0.028 | - | - | - |
| Variovorax | 0 | 0 | CHD | 2.156 | 0.005 | - | - | - |
| Paludibacter | 0 | 0 | CHD | 2.104 | 0.021 | - | - | - |
| Howardella | 0 | 0 | CHD | 2.019 | 0.001 | - | - | - |

Results from LEfSe and MaAsLin together with the median abundance of each genera were presented, sorting firstly by group and then, by LDA score. Only genera that had an LDA score > 2 at a Q_FDR_ < 0.05 from LEfSe were shown and selected for further analysis by MaAsLin. In the MaAsLin analyses, age, BMI, ethnicity, residence, alcohol consumption, negative life events, and dietary intake were used as covariates. For results of MaAsLin analysis, only genera with a Q_FDR_ < 0.05 were shown.

BMI, body mass index; CHD, congenital heart disease; LDA, line discriminant analysis; MCHD, mothers of infants with CHD; MCG, mothers of infants without CHD;

**Supplementary Table 5 Differentially expressed plasma metabolites identified by univariate and multivariate analyses**.

| Metabolites | Characteristics | | Fold change | Mean abundance | | OPLS-DA | T-test | Linear regression | RDS of QC samples (%) |
| --- | --- | --- | --- | --- | --- | --- | --- | --- | --- |
|  | m/z | rt(s) |  | MCHD | MCG | VIP | Q_FDR_ | p-value |  |
| 1-Myristoyl-sn-glycero-3-phosphocholine | 468.3 | 364.4 | 1.786 | 25342.9 | 13732.7 | 2.041 | < 0.001 | **< 0.001** | 8.9 |
| Chenodeoxycholate | 451.3 | 285.4 | 1.729 | 36606.2 | 18790.6 | 1.948 | 0.003 | **0.013** | 34.3 |
| Uracil | 111.0 | 162.3 | 1.639 | 5094.2 | 2952.9 | 1.025 | < 0.001 | **< 0.001** | 47.9 |
| Glycerophosphocholine | 258.1 | 737.0 | 1.510 | 28846.0 | 18569.0 | 1.400 | < 0.001 | **< 0.001** | 11.1 |
| Hydrocortisone (Cortisol) | 421.2 | 87.3 | 1.441 | 21280.7 | 14438.9 | 1.809 | < 0.001 | **< 0.001** | 8.1 |
| L-Pipecolic acid | 147.1 | 1012.5 | 1.439 | 17256.6 | 11487.6 | 1.086 | < 0.001 | **< 0.001** | 5.6 |
| 1-Palmitoyl-sn-glycero-3-phosphocholine | 518.3 | 356.5 | 1.433 | 29343.2 | 19799.8 | 1.715 | < 0.001 | **< 0.001** | 14.5 |
| Dioctyl phthalate | 391.3 | 59.9 | 1.413 | 27025.1 | 18961.3 | 1.324 | < 0.001 | **0.002** | 27.5 |
| 1-Stearoyl-sn-glycerol 3-phosphocholine | 523.4 | 337.7 | 1.412 | 27137.5 | 18765.1 | 1.741 | < 0.001 | **< 0.001** | 7.5 |
| 1-Oleoyl-sn-glycero-3-phosphocholine | 522.4 | 337.7 | 1.405 | 98312.7 | 68267.7 | 3.285 | < 0.001 | **< 0.001** | 9.3 |
| L-Palmitoylcarnitine | 400.3 | 314.8 | 1.391 | 12802.7 | 9032.7 | 1.052 | < 0.001 | **< 0.001** | 11.8 |
| Hydroxyisocaproic acid | 131.1 | 260.7 | 1.367 | 19724.2 | 14263.5 | 1.374 | 0.001 | **0.001** | 40.4 |
| Norethindrone Acetate | 339.2 | 52.3 | 1.220 | 322565.7 | 261014.0 | 4.716 | 0.004 | **0.005** | 10.6 |
| 2-Hydroxy-3-methylbutyric acid | 117.1 | 265.0 | 1.219 | 19719.7 | 15614.1 | 1.191 | 0.005 | **0.003** | 33.1 |
| Myristoleic acid | 225.2 | 86.3 | 1.212 | 65586.0 | 52838.1 | 2.444 | 0.019 | **0.012** | 9.4 |
| L-Pyroglutamic acid | 128.0 | 572.8 | 1.210 | 50725.3 | 41013.5 | 1.996 | 0.017 | **0.016** | 36.8 |
| L-Valine | 116.1 | 567.3 | 1.195 | 83993.9 | 68875.8 | 2.425 | < 0.001 | **< 0.001** | 7.4 |
| Choline | 104.1 | 491.7 | 1.192 | 150756.2 | 123327.3 | 2.266 | 0.001 | **0.002** | 38.6 |
| 1-Stearoyl-2-hydroxy-sn-glycero-3-phosphocholine | 568.3 | 344.7 | 1.190 | 60517.6 | 49801.5 | 1.634 | 0.004 | **0.001** | 12.1 |
| L-Leucine | 130.1 | 491.5 | 1.182 | 169807.9 | 141032.3 | 2.620 | 0.001 | **< 0.001** | 11.9 |
| D-Proline | 116.1 | 586.8 | 1.166 | 47516.6 | 40638.5 | 1.149 | 0.015 | 0.780 | 13.4 |
| Uridine | 243.1 | 301.7 | 1.165 | 32934.5 | 27533.7 | 1.488 | 0.001 | **< 0.001** | 3.2 |
| cis-9-Palmitoleic acid | 253.2 | 84.2 | 1.149 | 672906.8 | 572725.2 | 7.042 | 0.046 | **0.017** | 3.3 |
| L-Isoleucine | 130.1 | 515.6 | 1.148 | 91242.7 | 77865.4 | 1.629 | 0.006 | **0.007** | 5.4 |
| L-Tryptophan | 203.1 | 477.0 | 1.130 | 34841.2 | 30153.7 | 1.147 | 0.018 | **0.020** | 21.8 |
| L-Phenylalanine | 166.1 | 481.4 | 1.121 | 82519.4 | 71661.5 | 1.014 | 0.001 | **< 0.001** | 6.1 |
| Creatinine | 114.1 | 315.3 | 1.100 | 384536.5 | 340633.9 | 2.275 | < 0.001 | **< 0.001** | 7.2 |
| Betaine | 118.1 | 514.6 | 1.083 | 888080.6 | 794402.2 | 2.828 | 0.002 | **< 0.001** | 4.2 |
| 1-Palmitoylglycerol | 313.3 | 67.0 | 1.077 | 98827.1 | 90902.9 | 1.539 | 0.016 | **0.024** | 19.4 |
| Palmitic acid | 255.2 | 67.3 | 0.915 | 49622.3 | 52831.6 | 1.583 | 0.043 | **0.015** | 5.7 |
| Arachidonic Acid (peroxide free) | 303.2 | 79.2 | 0.883 | 276005.3 | 305970.2 | 3.099 | 0.016 | 0.162 | 10.9 |
| Nname,cis-9,10-Epoxystearic acid | 297.2 | 88.6 | 0.879 | 102194.4 | 114413.3 | 2.635 | < 0.001 | **0.013** | 7.6 |
| Thioetheramide-PC | 780.6 | 259.4 | 0.856 | 163557.6 | 187045.8 | 3.470 | 0.020 | 0.108 | 29.3 |
| 2E-Eicosenoic acid | 309.3 | 78.4 | 0.847 | 96521.3 | 110506.6 | 2.937 | 0.047 | 0.118 | 9.1 |
| D-Allose | 239.1 | 575.8 | 0.836 | 98204.3 | 114936.5 | 2.736 | 0.027 | 0.104 | 30.7 |
| Heptadecanoic acid | 269.2 | 79.3 | 0.832 | 49120.7 | 58019.6 | 2.051 | 0.001 | **0.001** | 13.8 |
| 16-Hydroxypalmitic acid | 271.2 | 127.5 | 0.830 | 39335.0 | 46429.0 | 1.991 | < 0.001 | **0.001** | 20.6 |
| Sunitinib | 397.2 | 61.0 | 0.815 | 92243.7 | 112619.8 | 2.597 | 0.015 | **0.042** | 6.9 |
| 1-Stearoyl-sn-glycerol | 359.3 | 65.8 | 0.815 | 60300.3 | 73218.6 | 2.400 | < 0.001 | **0.002** | 31.4 |
| PC(16:0/16:0) | 756.6 | 264.6 | 0.814 | 131711.5 | 158503.4 | 3.476 | 0.001 | **0.003** | 30.8 |
| Pristanic acid | 297.3 | 75.8 | 0.786 | 17746.9 | 22257.3 | 1.655 | < 0.001 | **< 0.001** | 9.0 |
| Alpha-D-Glucose | 179.1 | 577.7 | 0.785 | 326411.3 | 409607.8 | 7.515 | < 0.001 | **< 0.001** | 12.2 |
| D-Tagatose | 161.0 | 577.6 | 0.777 | 17673.9 | 22253.2 | 1.736 | < 0.001 | **< 0.001** | 14.0 |
| Eicosapentaenoic acid | 301.2 | 79.9 | 0.759 | 32452.8 | 41923.3 | 1.589 | < 0.001 | **0.003** | 12.3 |
| D-Mannose | 198.1 | 574.9 | 0.753 | 35012.5 | 45918.1 | 2.084 | < 0.001 | **< 0.001** | 31.9 |
| Methylmalonic acid | 117.0 | 195.6 | 0.732 | 21108.1 | 28526.1 | 1.549 | 0.003 | **0.005** | 7.0 |
| alpha-Tocopherol (Vitamin E) | 430.4 | 60.2 | 0.720 | 7724.4 | 10377.4 | 1.034 | 0.001 | **0.043** | 41.9 |
| m-Chlorohippuric acid | 213.0 | 180.3 | 0.701 | 29302.2 | 40972.4 | 2.677 | < 0.001 | **< 0.001** | 17.8 |
| Acetylcarnitine | 204.1 | 576.3 | 0.694 | 84937.5 | 117035.6 | 3.381 | 0.001 | **0.007** | 5.9 |
| 1-Stearoyl-2-oleoyl-sn-glycerol 3-phosphocholine (SOPC) | 832.6 | 253.3 | 0.666 | 53562.2 | 80327.3 | 3.505 | < 0.001 | **< 0.001** | 42.2 |
| Hypoxanthine | 135.0 | 314.2 | 0.650 | 15068.9 | 22882.3 | 1.517 | 0.001 | **0.008** | 4.4 |
| Acetoacetic acid | 101.0 | 192.7 | 0.648 | 14200.4 | 20892.6 | 1.304 | 0.001 | **0.015** | 8.8 |
| 1,2-dioleoyl-sn-glycero-3-phosphatidylcholine | 768.6 | 257.0 | 0.646 | 55486.3 | 83840.0 | 3.549 | < 0.001 | **< 0.001** | 13.4 |
| L-Gulonic gamma-lactone | 177.0 | 196.6 | 0.635 | 222890.2 | 343746.2 | 8.187 | < 0.001 | **< 0.001** | 7.7 |
| Sphingomyelin (d18:1/18:0) | 794.6 | 254.2 | 0.614 | 69918.6 | 111244.6 | 4.300 | < 0.001 | **< 0.001** | 47.7 |
| Vanillin | 151.0 | 72.0 | 0.599 | 13316.8 | 21784.0 | 2.466 | < 0.001 | **< 0.001** | 17.5 |
| D-Ribose | 149.0 | 195.6 | 0.594 | 48563.9 | 79301.8 | 4.307 | < 0.001 | **< 0.001** | 6.6 |
| D(-)-beta-hydroxy butyric acid | 103.0 | 462.8 | 0.490 | 20455.3 | 41890.2 | 2.804 | 0.001 | **0.032** | 6.0 |

Results from OPLS-DA, fold change analysis, t-test, and linear regression, together with the mean abundance and characteristics of each metabolites were presented, sorting by the value from fold change analysis. Only the metabolites with a Q_FDR_ (FDR corrected p-value) < 0.05 from the t-test were selected for regression analysis. For regression analysis, age, BMI, ethnicity, residence, alcohol consumption, negative life events, and dietary intake were used as covariates; significant p-values (p < 0.05) are shown in bold.

BMI, body mass index; CHD, congenital heart disease; MCHD, mothers of infants with CHD; MCG, mothers of infants without CHD; OPLS-DA, supervised orthogonal partial least squares-discrimination analysis; QC, quality control; VIP, variable importance for the projection.

**Supplementary File 1 Definitions.**

**Gut microbiota**

The gut microbiota refers collectively to the microbial composition in the gut and includes approximately 1,014 species of bacteria that normally reside in the gastrointestinal tract, reaching a microbial cell number that far exceeds the number of human cells of the body.

**Metabolomics**

Metabolomics is a research strategy that applies high-throughput analysis techniques to conduct comprehensive qualitative and quantitative analysis of metabolites produced by cells or organisms under certain physiological periods or conditions (e.g., different pathological status). The analyzed object of metabolomics is the low molecular weight compounds secreted by organisms, mainly including amino acids, peptides, cholesterol, lipids, and carbohydrates.

**16S rRNA gene sequencing**

16S rRNA/16S ribosomal RNA is a component of the 30S subunit in the ribosome of prokaryotes. 16S rRNA gene sequencing provides information about the composition of microbial communities. With this approach, polymerase chain reaction is used to amplify a specific region of the 16S gene; the product is subsequently sequenced.
